# Modeling the impact of MRI acquisition bias on structural connectomes: Harmonizing structural connectomes

**DOI:** 10.1162/netn_a_00368

**Published:** 2024-10-01

**Authors:** Jagruti Patel, Mikkel Schöttner, Anjali Tarun, Sebastien Tourbier, Yasser Alemán-Gómez, Patric Hagmann, Thomas A. W. Bolton

**Affiliations:** Department of Radiology, Lausanne University Hospital and University of Lausanne (CHUV-UNIL), Lausanne, Switzerland

**Keywords:** Neuroimaging, Human Connectome Project, Diffusion MRI, Acquisition bias, Harmonization, Fingerprinting

## Abstract

One way to increase the statistical power and generalizability of neuroimaging studies is to collect data at multiple sites or merge multiple cohorts. However, this usually comes with site-related biases due to the heterogeneity of scanners and acquisition parameters, negatively impacting sensitivity. Brain structural connectomes are not an exception: Being derived from T1-weighted and diffusion-weighted magnetic resonance images, structural connectivity is impacted by differences in imaging protocol. Beyond minimizing acquisition parameter differences, removing bias with postprocessing is essential. In this work we create, from the exhaustive Human Connectome Project Young Adult dataset, a resampled dataset of different *b*-values and spatial resolutions, modeling a cohort scanned across multiple sites. After demonstrating the statistical impact of acquisition parameters on connectivity, we propose a linear regression with explicit modeling of *b*-value and spatial resolution, and validate its performance on separate datasets. We show that *b*-value and spatial resolution affect connectivity in different ways and that acquisition bias can be reduced using a linear regression informed by the acquisition parameters while retaining interindividual differences and hence boosting fingerprinting performance. We also demonstrate the generative potential of our model, and its generalization capability in an independent dataset reflective of typical acquisition practices in clinical settings.

## Author Summary

One of the main roadblocks to using multisite neuroimaging data is the effect of acquisition bias due to the heterogeneity of acquisition parameters associated with various sites. This can negatively impact the sensitivity of machine learning models employed in neuroscience. Thus, it is extremely important to model the effect of this bias. In this work, we address this issue at the level of brain structural connectivity, an important biomarker for various brain disorders. We propose a simple linear regression model to minimize this effect using high-quality data from the Human Connectome Project, and show its generalizability to a clinical dataset.

## INTRODUCTION

Neuroimaging deals with different imaging techniques to study the structure and function of the brain (Zhang et al., 2020). The study of the brain with T1-weighted (T1W) and diffusion magnetic resonance imaging (MRI) has spurred the discovery of biomarkers reflective of brain pathophysiology (Garcia-Alloza & Bacskai, 2004; Schwarz, 2021). For example, the decrease in gray matter volume estimated using T1W MRI is an important biomarker for brain aging/disease (Schwarz, 2021), and abnormal changes in diffusion-derived maps like fractional anisotropy or mean diffusivity have been associated with neurological diseases such as multiple sclerosis and cerebral small vessel disease (Croall et al., 2017; Martinez-Heras, Grussu, Prados, Solana, & Llufriu, 2021).

While T1W and diffusion MRI have shown their individual potential for biomarker discovery, with the advent of tractography (Basser, Pajevic, Pierpaoli, Duda, & Aldroubi, 2000; Conturo et al., 1999) their combination has opened the door to yet other perspectives. Indeed, when one defines gray matter regions of interest from T1W MRI and connections with white matter tracts linking them using diffusion-weighted imaging (DWI), a map of physical wiring between brain regions, the so-called *structural connectome* (SC), can be constructed (Contreras, Goñi, Risacher, Sporns, & Saykin, 2015; Hagmann, 2005; Hagmann et al., 2007). The SC provides novel perspectives on brain diseases that could not have been unraveled from each modality alone (Wu et al., 2021). Additionally, graph metrics reflecting higher order properties of SCs (Rubinov & Sporns, 2010) can also serve as important diagnostic and prognostic biomarkers of cognition and neurological diseases: For instance, higher segregation and lower integration are found in traumatic brain injury patients or obsessive-compulsive disorder (Baldi et al., 2022; Imms et al., 2019).

For SC-related findings to be clinically useful, they must exhibit high generalizability. However, this is complicated by the small cohort size considered in most studies (Orban et al., 2018; Pinto et al., 2020). While statistical power and generalizability can be increased using large-scale multisite/longitudinal neuroimaging studies, biases arising owing to heterogeneity in equipment or acquisition parameters (APs) across sites/over time (Yu et al., 2018) must be controlled. Acquisition biases such as changes in scanner manufacturer, hardware/software of the scanner, or APs (e.g., voxel size and *b*-values) have all been pinpointed as sources of bias (Panman et al., 2019; Zhu, Moyer, Nir, Thompson, & Jahanshad, 2019), whose deleterious impacts also extend to SC-derived graph metrics (Borrelli, Cavaliere, Salvatore, Jovicich, & Aiello, 2022; Caiazzo et al., 2018; Messaritaki, Dimitriadis, & Jones, 2019).

Further downstream, the SC is also impacted by the parameters specified for parcellation and tractography (Petrov et al., 2017; Sotiropoulos & Zalesky, 2019; Yeh, Jones, Liang, Descoteaux, & Connelly, 2021). For parcellation, larger regions may entail averaging over inhomogeneities, whereas smaller regions could increase computational burden (Sotiropoulos & Zalesky, 2019). For tractography, the tracking of white matter tracts using DWI depends on the choice of diffusion encoding scheme (Gigandet et al., 2013), diffusion reconstruction models, streamline methods (deterministic vs. probabilistic), number of streamlines, and yet other preprocessing choices (Borrelli et al., 2022; Petrov et al., 2017; Roine et al., 2019; Yeh et al., 2021), complicating the reproducibility of tractography studies across research groups using different algorithms (K. G. Schilling et al., 2019). Even after SC construction, further choices such as defining edge weights, or considering weighted versus binary or sparse versus dense networks, can exert additional impacts on extracted graph metrics (Borrelli et al., 2022; Messaritaki et al., 2019; Roine et al., 2019; Sotiropoulos & Zalesky, 2019; Yeh et al., 2021).

To alleviate the issues caused by the plethora of existing sources of bias, data harmonization has been heavily studied in neuroimaging. Harmonizing as much as possible the scanning protocols and using ideally similar machines across different sites is essential (George, Kuzniecky, Rusinek, Pardoe, & Human Epilepsy Project Investigators, 2020; Gountouna et al., 2010; Vavasour et al., 2019). However, a perfect match between sites is rarely possible. Hence, full elimination of scanner bias at acquisition is impossible, except in the rare situations where all sites use the same machines and same parameters (Bordin et al., 2021; Jovicich et al., 2016; Noble et al., 2017; Wittens et al., 2021; Yu et al., 2018), or even more conservatively, the same hardware/software version from the same vendor (Shinohara et al., 2017). Even though such an effort may alleviate the problem of planning a large-scale multisite data collection, it does not solve the problem of harmonizing already existing data collected at different sites, with different scanners, different protocols, and imaging parameters, and at different time points (Craddock et al., 2013; Sudlow et al., 2015; Toro et al., 2018; Van Essen et al., 2012). For taking advantage of such heterogeneous data, the acquisition effect must be removed or modeled, either at the level of MRI data or on subsequently derived metrics, before analysis.

One of the simplest modeling methods is linear regression, where scanner bias can be modeled as a covariate either as a fixed effect (Chua et al., 2015) or a random effect (Kim et al., 2022). In past works, linear correction factors reduced scanner bias in diffusivity metrics or structural features (Kim et al., 2022; Venkatraman et al., 2015), and the approach was effective in correcting scanner bias while preserving the effects of diseases like schizophrenia and major depressive disorder (Nakano et al., 2020; Rozycki et al., 2018). While most studies have used linear regression as a statistical tool, Rozycki et al. (2018) have explored its use as a machine learning framework, where the parameters obtained from a training sample can be applied to an independent dataset.

The most commonly applied type of harmonization is the batch correction tool ComBat (Johnson, Li, & Rabinovic, 2007), which models additive and scaling effects of site bias at the level of the metrics at hand, and thus removes site effects in mean and variance while accounting for other desirable factors (e.g., clinical diagnosis) through dedicated covariates. ComBat successfully harmonizes SCs (Onicas et al., 2022) as well as many other brain metrics (Bostami, Calhoun, Van Der Horn, & Vergara, 2021; Fortin et al., 2017, 2018; Ingalhalikar et al., 2021; Radua et al., 2020). Modifications of the original model have been proposed to enable, for instance, the additional removal of site effects for covariance (Chen et al., 2022), the modeling of nonlinear impacts of some covariates (Pomponio et al., 2020; Sun et al., 2022), more flexible centering to the location and scale of a predetermined reference (Da-Ano et al., 2020), or the handling of multiple imaging parameters at a single site rather than a single batch effect (Carré et al., 2022; Horng et al., 2022). Furthermore, while conventional ComBat, being a statistical method, cannot be used to harmonize unseen data, some alternatives are capable of learning the harmonization parameters on a training set and then applying them to new test cases (Da-Ano et al., 2021; Garcia-Dias et al., 2020).

While existing methods can remove site bias, they suffer from specific limitations and, thus, work optimally in specific settings. For example, a generalized linear model with *traveling subjects* (TS, subjects who are scanned in more than one site) exhibited better performance than ComBat in studies when the sample size was small (20 subjects) (Itahashi et al., 2021; Maikusa et al., 2021), but Roffet, Delrieux, and Patow (2022) showed that a larger fraction of the site effect could be removed with ComBat. ComBat shows subpar performance when the sites have unbalanced datasets of patients and healthy controls, requiring TS to separate the measurement bias from the biological sampling bias (Yamashita et al., 2019). Furthermore, how it fares compared with both a simpler site-wise demeaning (Reardon, Li, & Hu, 2021) or more complex deep learning models (Cackowski, Barbier, Dojat, & Christen, 2021) is still debated. In short, it is thus crucial to select a harmonization method based on the sought clinical application (Gebre et al., 2023). Furthermore, there is evident value in considering simple linear techniques in exploratory attempts to remove the scanning bias from SCs.

In this work, we introduce a different data harmonization approach using linear regression. Compared with previous conceptually related work (Chua et al., 2015; Kim et al., 2022; Nakano et al., 2020; Rozycki et al., 2018; Venkatraman et al., 2015), we specifically focus on the harmonization of the SCs (at the level of individual connections), as well as of subsequently extracted graph theoretical metrics reflective of integration and segregation (regional level). Instead of treating scanner bias as an individual covariate, we enable its dissociation into several physically relevant factors. Feeding information regarding these factors to the harmonization model might serve as valuable prior knowledge to improve performance (Borges et al., 2019). In the present context, maximal *b*-value of DWI acquisitions and the spatial resolution of the diffusion images are the most relevant APs and are the focus of our study. We assess the efficacy of our approach with data from the Human Connectome Project Young Adult (HCP-YA) dataset, a benchmark dataset including T1W as well as diffusion images for over 1,000 healthy subjects (Glasser et al., 2013; Sotiropoulos et al., 2013; Van Essen et al., 2012, 2013), often leveraged in past studies to probe the impacts of various parameters on SCs and the associated graph metrics (Borrelli et al., 2022; Messaritaki et al., 2019; Roine et al., 2019).

Rather than diagnostic category, as in most existing contributions, we set to investigate the ability of our method to identify interindividual variability; that is, to fingerprint individual participants across scans acquired at different parameter combinations. First, we conduct an in-depth analysis of the impact of *b*-value and spatial resolution on SCs and SC-derived graph metrics. We show that the differences caused by these factors are efficiently removed by our harmonization approach and that it lowers the distance between scans from the same subject below the intersubject distance, regardless of the exact model parameters, which indicates the feasibility of fingerprinting. We then confirm the ability to fingerprint subjects in an independent test-retest dataset and exemplify the ability of our model to infer structural connectivity at other unseen AP values. Additionally, we validate the fingerprinting potential of our model on a fully independent dataset acquired in the context of a clinical study, harmonizing SCs obtained with two different diffusion imaging acquisitions and distinct *b*-values.

## METHODS

### Dataset

**Figure 1.**
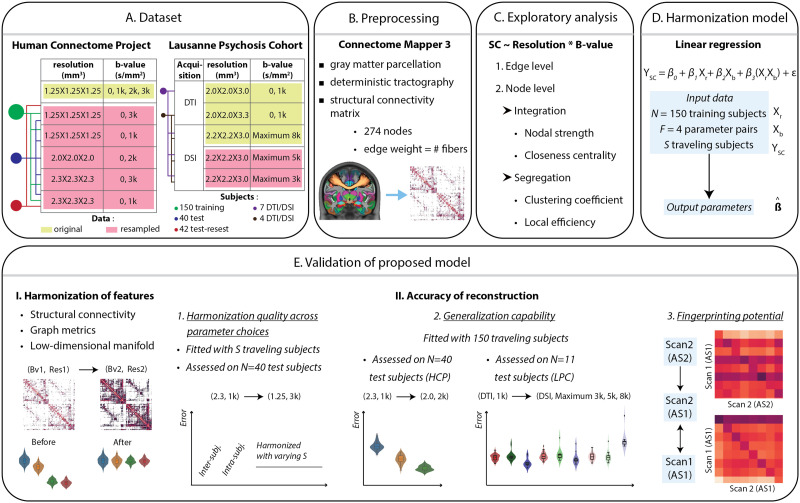
Schematic summary of the study. (A) Human Connectome Project (HCP) diffusion-weighted images with an original spatial resolution of 1.25 mm isotropic were the basis of the dataset that was resampled with isotropic voxel spacing of 1.25/2.3 mm for the training/test-retest (TRT) set, and 1.25/2.0/2.3 mm for the test set; *b*-value (*bval*) of 1,000/3,000 s/mm^2^ for the training/TRT set, and 1,000/2,000/3,000 s/mm^2^ for the test set. Eleven healthy subjects selected from Lausanne Psychosis Cohort (LPC) and containing diffusion tensor imaging (DTI) and diffusion spectrum imaging (DSI) acquisitions were used as an independent dataset for validation purposes. DTI images with *bval* of 1,000 s/mm^2^ were available at a spatial resolution of 2 × 2 × 3 mm^3^ (7 subjects) and 2 × 2 × 3.3 mm^3^ (4 subjects). DSI images with an original maximum *bval* of 8,000 s/mm^2^ were available at a spatial resolution of 2.2 × 2.2 × 3 mm^3^ for all the subjects. The DSI images were then resampled to have maximum *bval* as a factor of variation (3,000, 5,000 s/mm^2^). (B) For each subject, a structural connectome (SC) with 274 brain regions was constructed through state-of-the-art preprocessing (brain slice reproduced from Alemán-Gómez et al., 2022). (C) Each individual structural connection was analyzed in a two-way ANOVA to assess the respective impacts of *bval* and spatial resolution (*res*), as well as their interaction. A similar process was applied to selected graph theoretical metrics reflective of integration and segregation. (D) SC harmonization was performed with a linear regression model accounting for the effects of *bval*, *res*, and their interaction. (E) Harmonization quality was probed on individual connections, graph theoretical metrics, and in a low-dimensional representation of the data. The impact of model parameters (number of training subjects, use of traveling subjects) was assessed, on test subjects, through distances between high-quality SCs (*res* = 1.25, *bval* = 3,000) and harmonized low-quality SCs (*res* = 2.3 to 1.25, *bval* = 1,000 to 3,000), as compared with the distance across scans without harmonization (*Intra-subj.*), or to the distance across subjects for high-quality SCs (*Inter-subj.*). To gauge the generalizability of the model, on test subjects from the HCP, low-quality data were further harmonized to *res* = 2, *bval* = 2,000 and compared with the SCs obtained at these acquisition parameter values. On test subjects from the LPC, DTI-derived SCs at *bval* 1,000 were harmonized to DSI SCs at maximum *bval* of 3,000, 5,000, and 8,000 (using our model trained on the HCP data) and compared with the actual DSI-derived SCs at these maximum *bval* settings. Fingerprinting potential was assessed on TRT subjects as well as 11 LPC subjects, before or after harmonizing the second scan obtained with acquisition settings 2 (AS2) to the first scan (obtained with acquisition settings 1, AS1).

Figure 1 presents a schematic overview of the different steps included in our study. The data we used (Figure 1A) included the minimally preprocessed T1W and diffusion-weighted MRI scans of 190 subjects from the HCP-YA dataset, randomly partitioned into 150 training subjects and 40 test subjects, as well as the minimally preprocessed scans of 44 independent test-retest (TRT) subjects, for whom two scans were available. For these 44 subjects, the scan-rescan time interval was in the range of 18–343 days. One TRT subject was removed from the analyses because of problems within preprocessing upon quality control. One TRT subject was removed at the level of the analyses, as the inter-scan difference for this subject at the level of SCs acquired at the same APs was on par with inter-scan intersubject differences.

All three groups had subjects within the age range of 22–36 and the male/female ratio was 60/90, 24/16, and 12/30 for training, test, and test-retest groups, respectively. In supplementary analyses, we verified that our results were not affected by gender bias (data not shown). The data originally included T1W images at 0.7 × 0.7 × 0.7 mm^3^ spatial resolution, and diffusion-weighted images acquired at *b*-values of 0, 1,000, 2,000, and 3,000 s/mm^2^ (approximately equal number of directions for each nonzero *b*-value and 18 b0 images; Borrelli et al., 2022) with a spatial resolution of 1.25 × 1.25 × 1.25 mm^3^. For computational reasons, the original T1W data were downsampled to isotropic voxel spacing of 1.25 mm before any further preprocessing. Furthermore, only one *b*-value (1,000, 2,000, or 3,000 s/mm^2^) was used together with the null reference *b*-value (0 s/mm^2^) when generating a given SC; all *b*-values between 0 and 15 s/mm^2^ were regarded as zero *b*-value and similarly, for nonzero *b*-values, any value in the range of ±20 s/mm^2^ from that *b*-value was considered to be that *b*-value.

**Figure 2.**
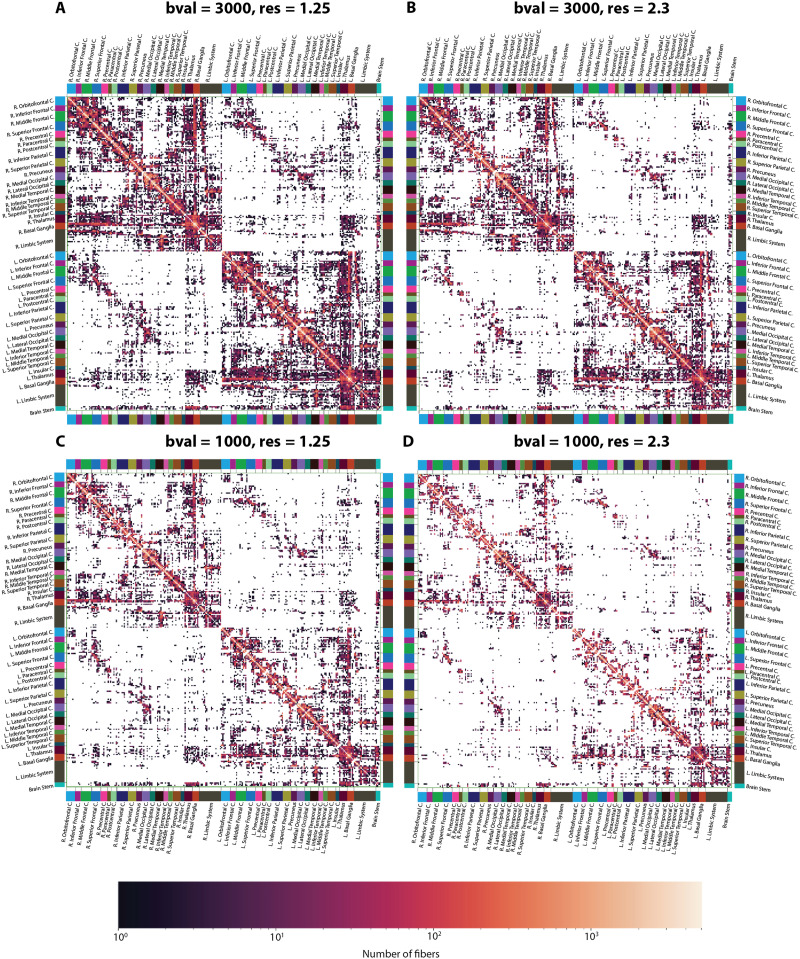
Structural connectome of an indicative subject at four different acquisition parameters before harmonization. Structural connectivity of the subject at (A) *bval* = 3,000, *res* = 1.25, (B) *bval* = 3,000, *res* = 2.3, (C) *bval* = 1,000, *res* = 1.25, (D) *bval* = 1,000, *res* = 2.3. Each element in this matrix represents the number of fibers connecting two regions (nodes) of the brain where the darker the color, the larger the number of fibers. White signifies the absence of connection between a pair of brain regions.

**Figure 3.**
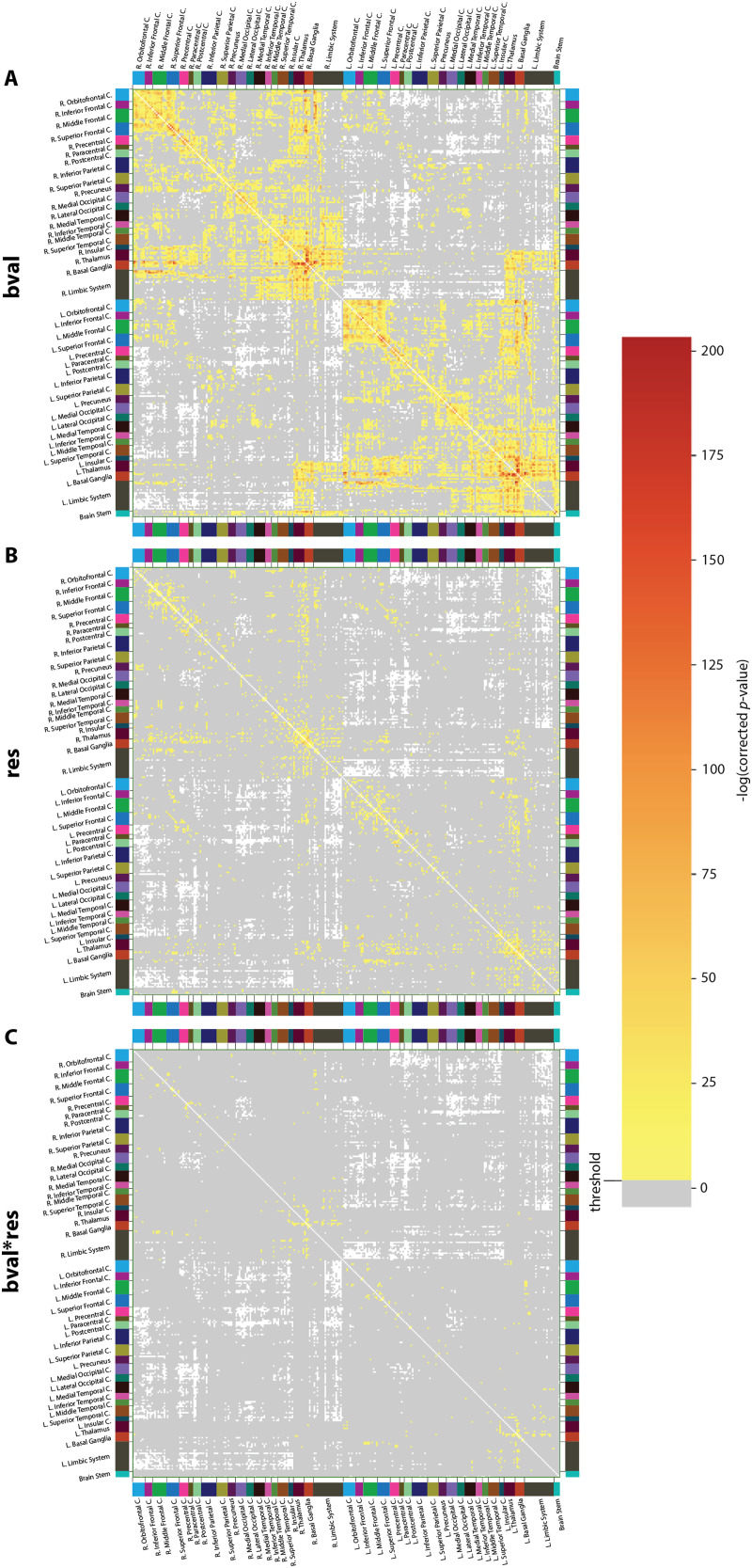
Impact of acquisition parameters on structural connectivity. Results of two-way ANOVA in terms of the impact of (A) *b*-value, (B) spatial resolution, and (C) interaction between these acquisition parameters (APs) at the level of connections. The ANOVA analysis was done independently on each of the connections across the entire dataset (training and test) of 190 subjects for the four AP combinations. Each value in the matrix is the negative of the log of the Bonferroni-corrected *p* values (*p* value × 37,401, where 37,401 is the number of possible connections). Any value lower than or equal to the threshold (−log(0.05)) is insignificant (gray in color). Any value greater than this threshold is significant, and the redder the color, the larger the significance of the bias on a connection. White signifies the absence of connection between a pair of brain regions.

**Figure 4.**
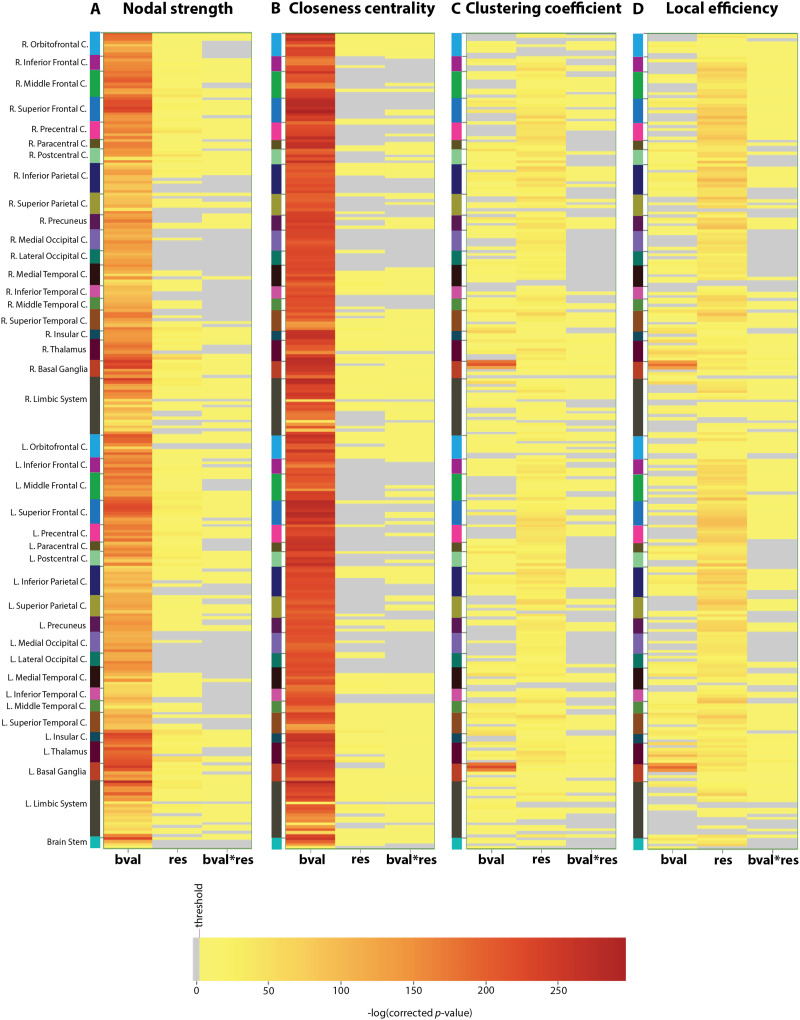
Impact of acquisition parameters on nodal metrics extracted from whole-brain structural connectivity. Results of two-way ANOVA in terms of the impact of *b*-value (*bval*), spatial resolution (*res*), and interaction between these aquisition parameters (APs; *bval* * *res*) on (A) nodal strength, (B) closeness centrality, (C) clustering coefficient, and (D) local efficiency. The ANOVA analysis was done independently on each of the nodes for each of these metrics across the entire dataset (training and test) of 190 subjects for the four AP combinations. Each value in the matrix is the negative of the log of the Bonferroni-corrected *p* values (*p* value × 274, where 274 is the number of nodes). Any value lower than or equal to the threshold (−log(0.05)) is insignificant (gray in color). Any value greater than this threshold is significant, and the redder the color, the larger the significance of the bias on a node for the corresponding nodal metric.

**Figure 5.**
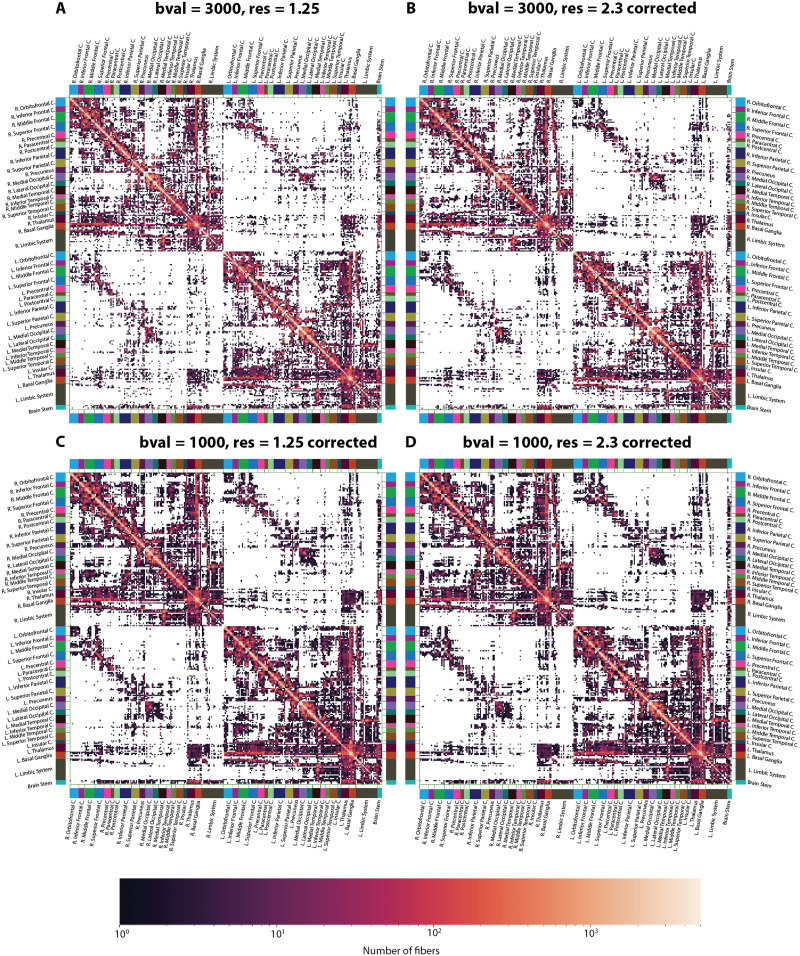
Structural connectome of an indicative subject after harmonization. Structural connectivity of the subject at (A) *bval* = 3,000, *res* = 1.25 (HBHR), (B) *bval* = 3,000, *res* = 2.3 corrected to HBHR, (C) *bval* = 1,000, *res* = 1.25 corrected to HBHR, (D) *bval* = 1,000, *res* = 2.3 corrected to HBHR. Each element in this matrix represents the number of fibers connecting two regions (nodes) of the brain where the darker the color, the larger the number of fibers. White signifies the absence of connection between a pair of brain regions.

**Figure 6.**
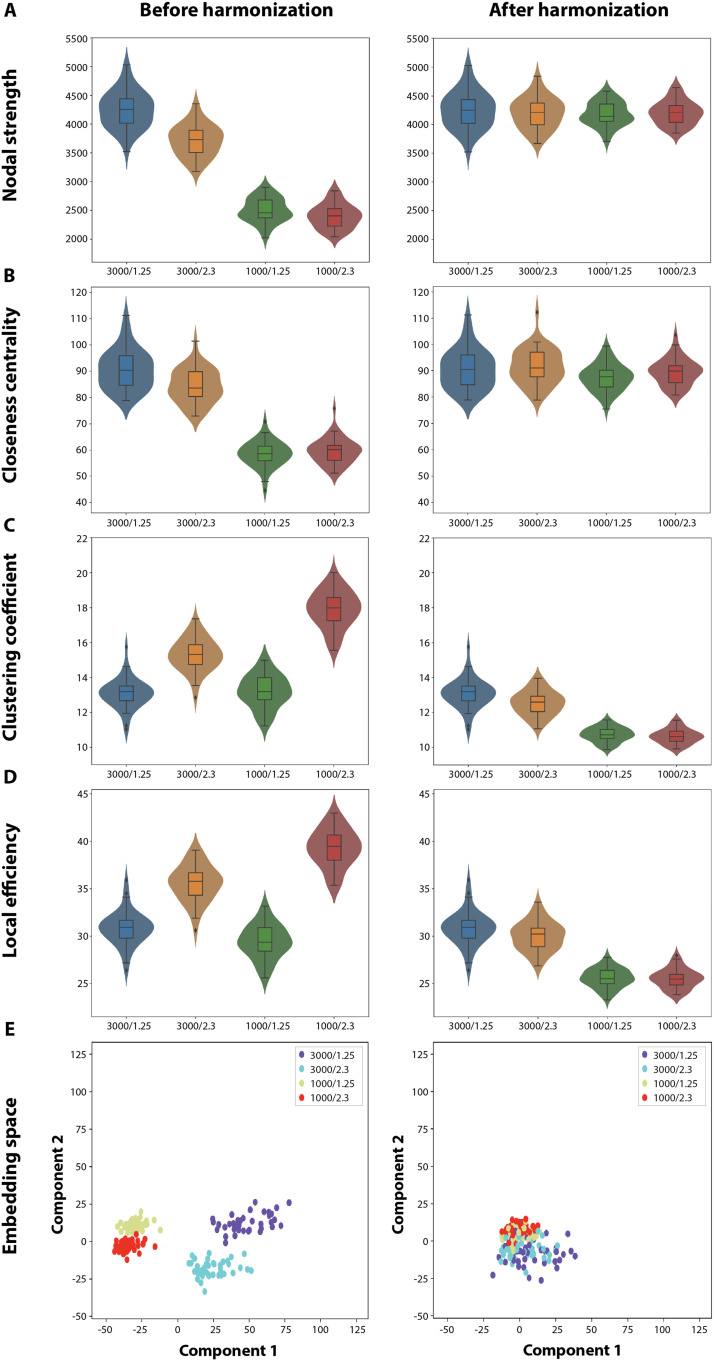
Effect of harmonization on connectivity metrics. The violin and box plots of the mean of the different nodal metrics (A) nodal strength, (B) closeness centrality, (C) clustering coefficient, and (D) local efficiency computed for the 40 test subjects across the four acquisition parameter (AP) combinations (3,000/1.25 for *b*-value (*bval*) = 3,000, spatial resolution (*res*) = 1.25, 3,000/2.3 for *bval* = 3,000, *res* = 2.3; 1,000/1.25 for *bval* = 1,000, *res* = 1.25; 1,000/2.3 for *bval* = 1,000, *res* = 2.3). (E) The embedding of the test subject structural connectomes (SCs) for the four AP combinations across the first two components obtained using principal component analysis. All the plots on the left are before harmonization and the ones on the right are after harmonization.

**Figure 7.**
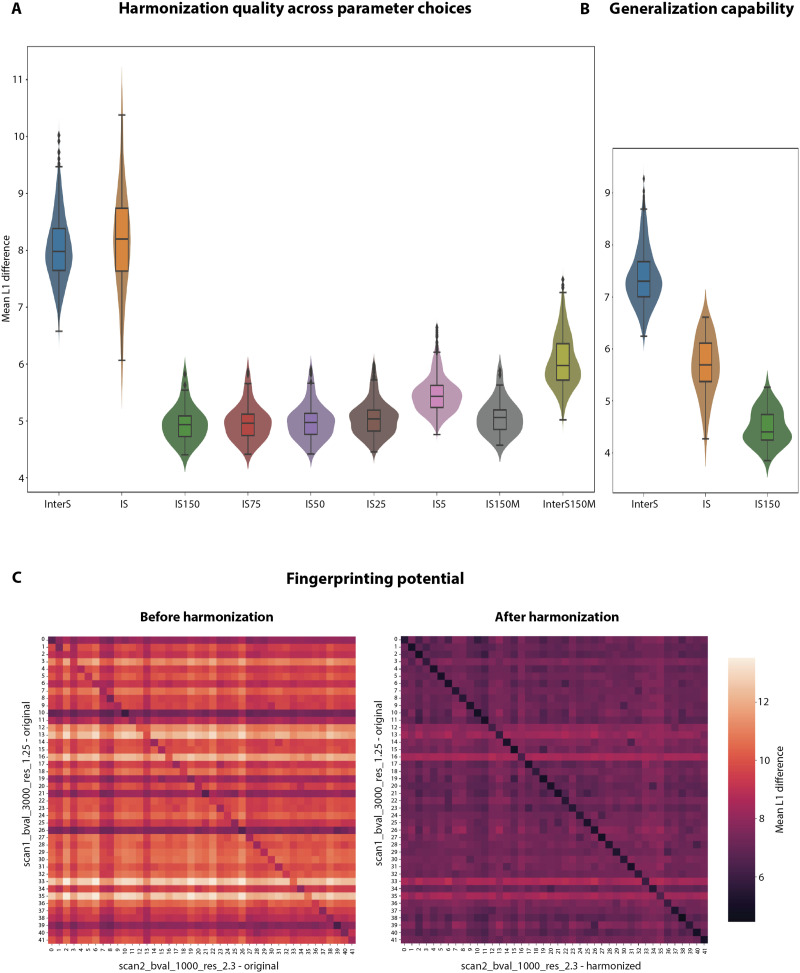
Accuracy of reconstruction—Human Connectome Project Young Adult Dataset. (A) The distribution of mean L1 differences across the 40 test subjects [at acquisition parameters (APs) (1.25, 3k), InterS] is compared with that within subjects across APs [(2.3, 1k) versus (1.25, 3k), IS], and to that within subjects when contrasting the (1.25, 3k) setting without harmonization to the one with harmonization from (2.3, 1k). Associated violins from left to right denote the use of 150 (IS150), 75 (IS75), 50 (IS50), 25 (IS25), or 5 (IS5) traveling subjects (TS) in model fitting (i.e., each contributing four data points), while the dark gray distribution (IS150M) reflects the use of 150 independent subjects (only one data point contributed by each). The rightmost distribution (InterS150M) depicts the intersubject differences after the SCs at APs (2.3, 1k) have been corrected to the (1.25, 3k) setting using 150 independent subjects in the model. (B) The distribution of mean L1 differences across test subjects [at APs (2.0, 2k), InterS] is compared with that within subjects across APs before harmonization [(2.0, 2k) versus (2.3, 1k), IS], and after harmonization [(2.0, 2k) versus (2.3, 1k) corrected using 150 TS in model fitting (i.e., each contributing four data points; IS150)]. (C) Distance matrices, with mean L1 difference as a metric, when Scan 2 and Scan 1 structural connectomes are compared for test-retest subjects before harmonization (left matrix), where Scan 1 is acquired at APs (1.25, 3k) while Scan 2 is acquired at (2.3, 1k), and after harmonization (right matrix), where Scan 1 is acquired at APs (1.25, 3k) while Scan 2 is acquired at (2.3, 1k) and corrected using 150 TS in model fitting (i.e., each contributing four data points). [Note: All the APs mentioned above are in the format (isotropic spatial resolution, *b*-value)].

To compare SCs generated at various APs, for training and test subjects, we considered resampled data with *b*-value of 1,000 or 3,000 s/mm^2^ (*bval* = 1,000 or 3,000) and spatial resolution of either isotropic spacing of 1.25 mm or 2.3 mm (*res* = 1.25 or 2.3; here, *res* is taken to be the cube root of the spatial volume of each voxel, as in subsequent sections we handle diffusion imaging data with non-isotropic voxels), for a total of four AP combinations. Diffusion images were downsampled using trilinear interpolation. These data are analyzed in Figures 2 to 7A. For the test subjects, a similar process was performed to generate data at *bval* = 2,000, *res* = 2 (data at intermediate, otherwise unseen, AP values). For TRT subjects as well, data were generated at *bval* = 1,000, *res* = 2.3 (“low-quality” data), and *bval* = 3,000, *res* = 1.25 (“high-quality” data). Associated results are presented in Figures 7B and 7C.

**Figure 8.**
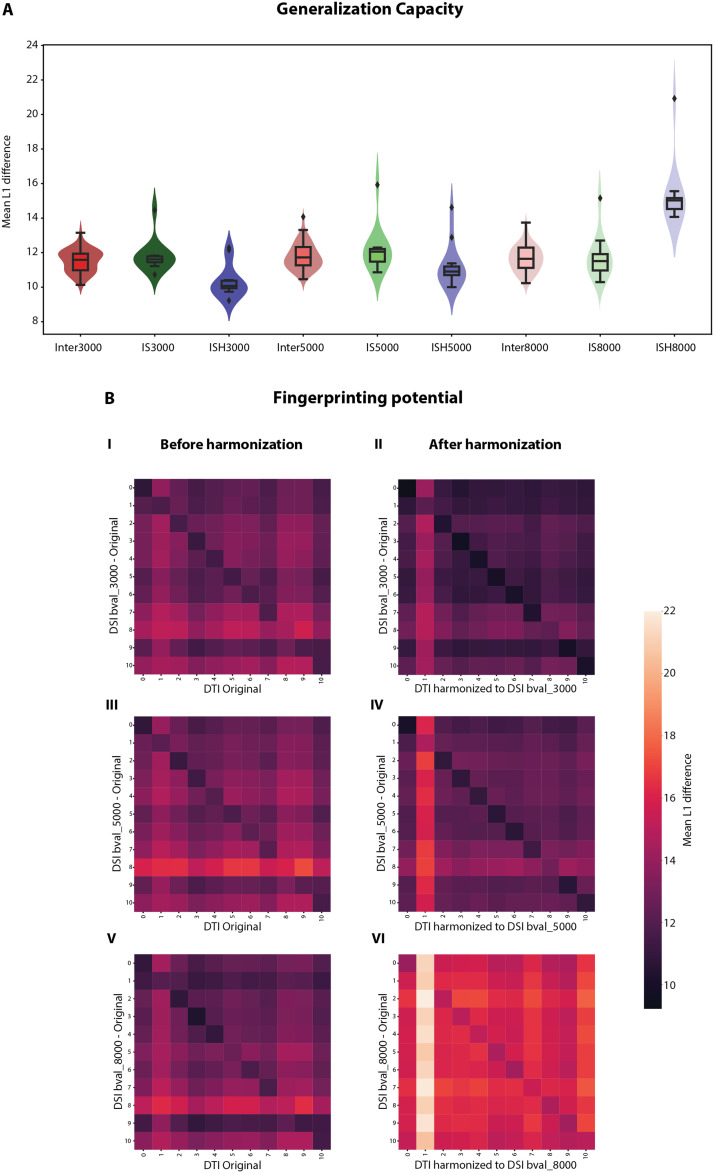
Accuracy of reconstruction—Lausanne Psychosis Cohort. (A) The distribution of mean L1 differences across the structural connectomes (SCs) of 11 subjects [acquired with diffusion spectrum imaging (DSI) at maximum *b*-value (*bval*) of 3,000 (Inter3000), 5,000 (Inter5000), or 8,000 (Inter8000)] is compared with that within subjects across the diffusion acquisitions before harmonization [DSI at maximum *bval* 3,000 versus diffusion tensor imaging (DTI) at *bval* 1,000; IS3000, and similar comparisons for DSI at maximum *bval* of 5,000, IS5000, and 8,000, IS8000] and after harmonization [DSI at maximum *bval* 3,000 versus DTI at *bval* 1,000 corrected using 150 Human Connectome Project Young Adult dataset traveling subjects in model fitting (i.e., each contributing four data points), ISH3000, and similar comparisons for DSI at maximum *bval* of 5,000, ISH5000, and 8,000, ISH8000]. (B) Distance matrices, with mean L1 difference as a metric, when SCs computed with DSI at maximum *bval* of 3,000 are compared against the DTI-derived SCs before harmonization (I) and after harmonization (II). Similar matrices for DSI at maximum *bval* of 5,000 (III, IV) and 8,000 (V, VI).

As a completely independent cohort, we also used unprocessed T1W, diffusion tensor imaging (DTI) (O’Donnell & Westin, 2011) and diffusion spectrum imaging (DSI) (Tian, Yan, & Zhang, 2009) data of 11 healthy subjects from the Lausanne Psychosis Cohort (Alemán-Gómez et al., 2023; Baumann et al., 2013, 2016; Griffa et al., 2019). The subjects were 19–54 years old, and two were females. The T1W images were acquired with 1-mm in-plane resolution and 1.2-mm slice thickness. The DTI images were acquired at *b*-values of 0 and 1,000 s/mm^2^ (30 directions for nonzero *b*-value with a spatial resolution of 2 × 2 × 3 mm^3^ for seven subjects and 36 directions for nonzero *b*-value with a spatial resolution of 2 × 2 × 3.3 mm^3^ for four subjects). The DSI images were acquired at a maximum *b*-value of 8,000 s/mm^2^ (128 diffusion-weighted directions and 1 b0 acquisition) with a spatial resolution of 2.2 × 2.2 × 3 mm^3^. The T1W data underwent N4 bias field correction (Tustison et al., 2010) and the DTI and DSI data underwent denoising, gibbs artifact removal, distortion correction, and eddy correction (Tax, Bastiani, Veraart, Garyfallidis, & Irfanoglu, 2022). After this, to keep the anatomical resolution constant, the preprocessed T1W data were downsampled to isotropic voxel spacing of 1.25 mm before any further processing. The preprocessed DSI data were resampled to include maximum *b*-value as a factor of variation (3,000, 5,000, and 8,000 s/mm^2^). Then, SCs were generated for each subject with the DTI acquisition and the three DSI acquisition variations. Associated results are presented in Figure 8.

### Preprocessing

The same preprocessing (Figure 1B) was applied for all scans and AP combinations. Connectome Mapper 3 v.3.0.0-rc4 (Tourbier et al., 2020, 2022) was used to construct the SCs. This software combines different processing pipelines and also provides the option to specify parameter settings for each preprocessing step. For segmentation of the T1W images, FreeSurfer recon-all version 6.0.1 was used, and parcellation of the cortical and subcortical surfaces was achieved using the Lausanne 2018 parcellation atlas (Cammoun et al., 2012; Desikan et al., 2006; Iglesias, Augustinack, et al., 2015; Iglesias, Van Leemput, et al., 2015; Najdenovska et al., 2018). In the present work, we considered the outputs at scale 3 (274 gray matter regions), as a good compromise between computational load, granularity of connectivity, and neurophysiological relevance of the parcels. The minimally preprocessed DWI data, already aligned to the T1w data, were used to generate tractograms using MRtrix (Tournier, Calamante, & Connelly, 2012), with the following parameters: constrained spherical deconvolution (CSD) of order 8, deterministic tractography with white matter seeding, and 10 million output streamlines.

Then, the aligned parcellated T1W image and tractograms were combined to generate SCs. Here, according to Borrelli et al. (2022), to minimize the known biases in fiber density, we selected the *number of fibers* as edge weight. Self-connections were set to zero.

For generating SCs from the above-preprocessed data of the Lausanne Psychosis Cohort, similar steps were followed except for a few changes. Instead of FSL’s flirt (Jenkinson, Beckmann, Behrens, Woolrich, & Smith, 2012), Advanced Normalization Tools (ANTs) (Avants, Epstein, Grossman, & Gee, 2008) were used for registering the T1W volume to the diffusion b0 image. Also, the fiber orientation distribution functions (ODFs) were estimated using MRtrix CSD of order 6 for the DTI data and Dipy (Garyfallidis et al., 2014) simple harmonic oscillator-based reconstruction and estimation (Özarslan, Koay, & Basser, 2008) of order 6 for the DSI data.

### Exploratory Analysis

#### Computation of graph theoretical metrics.

Selected graph metrics reflective of integration and segregation were calculated for the weighted undirected SCs for all 190 training and test subjects, for each AP combination. Our main analyses focused on nodal strength, closeness centrality, clustering coefficient, and local efficiency (Rubinov & Sporns, 2010). All metrics were computed using Networkx (Hagberg, Swart, & Schult, 2008).

Briefly, if *W* denotes the structural connectivity matrix for a given subject such that *w*_*ij*_ is each element in this matrix, nodal strength ($k_{i}^{w}$) for a node *i* is given by the following:

$$k_{i}^{w}=\Sigma_{j\in N,j\neq i}w_{ij},$$ (1)

where *N* is the set of all nodes in the network.

The clustering coefficient ($C_{i}^{w}$) for a node *i* is given by the following:

$$C_{i}^{w}=\frac{1}{k_{i}\left(k_{i}-1\right)}\Sigma_{j,h\in N,j,h\neq i}{\left(w_{ij}w_{ih}w_{jh}\right)}^{1/3},$$ (2)

where *k*_*i*_ is the number of neighbors (degree) of node *i*.

The other metrics require the computation of the distance matrix. Each element *l*_*ij*_ in the distance matrix *D* for a given subject is computed as follows:

$$l_{ij}=\left\{\begin{array}{ll} 1/w_{ij}, & \text{when}\;i\neq j\;\text{and}\;w_{ij}\neq 0.\\ 0, & \text{when}\;i=j. \end{array}\right.$$ (3)

This distance matrix *D* is required to calculate the shortest path length $d_{ij}^{w}$ from a node *i* to reach a node *j*.

The closeness centrality ($L_{i}^{w-1}$) for a node *i* is given by the following:

$$L_{i}^{w-1}=\frac{n-1}{\Sigma_{j\in N,j\neq i}d_{ij}^{w}},$$ (4)

where (*n* − 1) is the number of nodes reachable from node *i*.

The local efficiency ($E_{\left(loc\right)i}^{w}$) for a node *i* is given by the following:

$$E_{\left(loc\right)i}^{w}=\frac{1}{k_{i}\left(k_{i}-1\right)}\Sigma_{j,h\in N,j,h\neq i}{\left(w_{ij}w_{ih}{\left[d_{jh}^{w}\left(N_{-i}\right)\right]}^{-1}\right)}^{1/3},$$ (5)

where [$d_{jh}^{w}$(*N*_−*i*_)] is the shortest distance between nodes *j* and *h* in the subgraph formed by the neighbors of node *i* excluding *i*.

#### Effect of acquisition parameters.

In order to quantify the effects of *bval* and *res* (Figure 1C), a two-way ANOVA including interactions was applied to each edge of the SCs, either on the full set of 190 training and test subjects (Figure 3, Table 1A), or solely on the test subjects (Table 1B) for direct comparison to other conducted analyses. The edge-specific *p* value threshold was set to 0.05 divided by the maximal number of possible connections (37,401), which corresponds to a Bonferroni correction (Bonferroni, 1936).

**Table 1.** Effect of acquisition parameters on the structural connectivity and nodal metrics. Numbers of edges and nodes significantly impacted by the acquisition parameters (APs) *b*-value (*bval*), spatial resolution (*res*), and their interaction (*bval* * *res*) (A) on the entire dataset of 190 subjects for the four AP combinations before harmonization, and (B) on the test set of 40 subjects for the four AP combinations before and after harmonization. These are all the edges/nodes where the *p* value of the two-way ANOVA analysis is less than 0.05/37,401 (edges) or 0.05/274 (nodal strength, closeness centrality, clustering coefficient, local efficiency). The percentages of impacted edges and nodes are also displayed in parentheses, rounded off to the nearest integer, or, for those below 1, to two decimal places.

Number of subjects	**A**. Training + test = 190			**B**. Test = 40					
Harmonization status	Before harmonization						After harmonization		
APs	*bval*	*res*	*bval* * *res*	*bval*	*res*	*bval* * *res*	*bval*	*res*	*bval* * *res*
Graph features									
Edges (%)	9,012 (24)	1,992 (5)	330 (0.88)	2,889 (8)	151 (0.40)	2 (0.01)	172 (0.46)	72 (0.19)	91 (0.24)
Nodal strength (%)	274 (100)	218 (80)	167 (61)	274 (100)	118 (43)	15 (5)	0 (0)	0 (0)	0 (0)
Closeness centrality (%)	274 (100)	151 (55)	182 (66)	274 (100)	59 (22)	39 (14)	5 (2)	0 (0)	0 (0)
Clustering coefficient (%)	188 (69)	256 (93)	155 (57)	100 (37)	196 (72)	31 (11)	231 (84)	97 (35)	28 (10)
Local efficiency (%)	170 (62)	261 (95)	214 (78)	90 (33)	226 (82)	49 (18)	247 (90)	86 (31)	24 (9)

Similarly, a two-way ANOVA was also computed over the nodal metrics (nodal strength, closeness centrality, clustering coefficient, and local efficiency; Figure 4, Supporting Information Figure S1), and the nodes with a *p* value lower than 0.05/274 (the number of nodes) were considered significant (Table 1).

### Harmonization Model

In order to minimize the effects of scanner bias on the SCs (Figure 1D), the same process was similarly applied to each of the 37,401 connections at hand. Initially, the data of the 150 training subjects, across all four AP combinations (that is, a total of 600 data points), was used in the following linear regression model:

$$y_{i}=\beta_{0i}+\beta_{1i}\times X_{r}+\beta_{2i}\times X_{b}+\beta_{3i}\times X_{r}\times X_{b}+\varepsilon_{i},$$ (6)

where *y*_*i*_ is an estimated structural connection value (i.e., the number of fibers in our case), with *i* jointly indexing subjects and AP settings, [*β*_0*i*_, *β*_1*i*_, *β*_2*i*_, *β*_3*i*_]^⊤^ is the weight vector to be estimated for the connection at hand, and *X*_*r*_ and *X*_*b*_ are the *res* and *bval* values, respectively. In other words, a connection is simply modeled as a weighted linear combination of the APs and their interaction. This approach assumes that all the connections are independent of each other and that the errors (*ε*_*i*_) are random and independent, following a Gaussian distribution with a mean of zero. Also, subject-specific covariates are not explicitly modeled in order to be able to harmonize unseen data.

The model was trained on 150 traveling subjects, each with SCs for four APs, yielding a vector [$\hat{\beta }$_0*i*_, $\hat{\beta }$_1*i*_, $\hat{\beta }$_2*i*_, $\hat{\beta }$_3*i*_]^⊤^ of ordinary least squares estimates. Then, the structural connectivity of an unseen subject at one AP (*AP*_1_) was harmonized to another (*AP*_2_) as follows:

$$y_{i}^{\left({AP}_{2}\right)}=y_{i}^{\left({AP}_{1}\right)}+{\hat{\beta }}_{1i}\times \left(X_{r}^{\left({AP}_{2}\right)}-X_{r}^{\left({AP}_{1}\right)}\right)+{\hat{\beta }}_{2i}\times \left(X_{b}^{\left({AP}_{2}\right)}-X_{b}^{\left({AP}_{1}\right)}\right)+{\hat{\beta }}_{3i}\times \left(\left(X_{r}^{\left({AP}_{2}\right)}\times X_{b}^{\left({AP}_{2}\right)}\right)-\left(X_{r}^{\left({AP}_{1}\right)}\times X_{b}^{\left({AP}_{1}\right)}\right)\right).$$ (7)

Since the number of fibers should be a nonnegative integer, obtained values were rounded off to the nearest integer, and negative values were subsequently set to zero.

### Validation of Proposed Model

The validation of the harmonization model was done on the HCP-YA dataset and the Lausanne Psychosis Cohort. The harmonization of features was evaluated only on the HCP-YA dataset. However, accuracy of reconstruction in terms of generalization capability and fingerprinting potential was validated on both datasets.

#### Harmonization of features.

Assessment of differences before and after harmonization was conducted at the level of connections, regions, and in a low-dimensional space (Figure 1EI). All the AP combinations for the test subjects were harmonized to the higher *bval* higher *res* (HBHR) SCs (*bval* = 3,000 and *res* = 1.25). The SCs of an indicative test subject for all four AP combinations were plotted before and after harmonization, as shown in Figure 2 and Figure 5, respectively.

Graph metrics were calculated for the test set before and after harmonization. Histogram distributions were plotted for each of the nodal metrics for each AP combination across the test subjects (Supporting Information Figure S2). The mean of each nodal metric across regions, for each subject and AP combination, was calculated before and after harmonization. Then, violin and box plots were plotted for each of these mean nodal metrics (Figures 6A to 6D) for each AP combination across the test subjects.

Similarly to Da-Ano et al. (2021), in order to assess the impact of harmonization, the SCs were encoded in a low-dimensional space using principal component analysis (PCA; Figure 6E) (Hotelling, 1933; Pearson, 1901; Wold, Esbensen, & Geladi, 1987). Before harmonization, the test set was standardized with respect to the training set. Then, a PCA was fitted on the training set. Finally, the test set was projected onto the first two PCs. After harmonization, these steps were repeated with the harmonized training and test sets.

A two-way ANOVA was performed over the edges and the graph metrics for the test subjects after harmonization, and the numbers of significant edges and nodes were compared with those obtained before harmonization (Table 1B).

### Accuracy of Reconstruction

#### Harmonization quality across parameter choices.

In order to evaluate the model, HBHR SCs were predicted from lower *bval* lower *res* (LBLR) SCs (*bval* = 1,000 and *res* = 2.3) for the 40 test subjects. Then, the intrasubject differences (in the form of L1 differences averaged across the connections) were calculated between the actual HBHR and actual LBLR SCs (labeled *IS* in Figure 7A) or corrected LBLR SCs (labeled *IS150*). To check the performance of the model as a function of training set size, it was also trained with 75 subjects twice (on separate subsets), 50 subjects 3 times, 25 subjects 6 times, or 5 subjects 30 times, and subsequently evaluated on the test set similarly as above (respectively labeled *IS75*, *IS50*, *IS25*, and *IS5*). In addition, the model was trained under the constraint that no subject has been scanned twice, meaning that there were no TS (60 subjects at a *bval* of 3,000 and *res* of 1.25, and 30 subjects for each of the other AP combinations). This was also evaluated on the test set similarly as above (labeled *IS150M*). All these intrasubject errors were plotted against the pairwise mean absolute intersubject differences (labeled *InterS*) evaluated for actual HBHR SCs for the test subjects. Along with this, the pairwise mean absolute intersubject differences were also evaluated for the LBLR SCs corrected using the model without TS (*InterS150M*). In addition, a two-sample Kolmogorov-Smirnov (KS) test (Dodge, 2008) was conducted between the *InterS* and *IS* distributions, and between the *IS150M* and *InterS150M* distributions, to determine whether they were statistically different (*p* value < 0.05).

#### Generalization capability.

The original model with 150 TS was also tested, on the HCP-YA dataset, to generate SCs with APs not included in the training set. For the 40 test subjects, the SCs generated with *bval* of 1,000 and *res* of 2.3 were corrected to generate SCs with intermediate *bval* intermediate *res* (IBIR) (*bval* = 2,000 and *res* = 2.0). Then, the intrasubject differences (in the form of L1 differences averaged across the connections) were calculated between the actual IBIR and actual LBLR SCs (labeled *IS* in Figure 7B) or corrected LBLR SCs (labeled *IS150*). These intrasubject errors were plotted against the pairwise mean absolute intersubject differences (labeled *InterS*) evaluated for actual IBIR SCs for these test subjects. In order to validate the efficacy of our method, the accuracy and I_diff_ (Amico & Goñi, 2018) were calculated. In a pairwise mean absolute difference matrix, I_diff_ is defined as the difference between the average of the off-diagonal elements and the average of the diagonal elements; thus, it is the difference between the mean intersubject difference and the mean intrasubject difference.

Also, for checking the efficacy of our model in generalizing to independent datasets, DSI-derived SCs at maximum *bval* of 3,000, 5,000, and 8,000 were predicted from DTI-derived SCs at *bval* of 1,000 for the 11 subjects of the Lausanne Psychosis Cohort. Then, the intrasubject differences (in the form of L1 differences averaged across the connections) were calculated between the actual DSI-derived SCs for each of the maximum *bval* and actual DTI-derived SCs (labeled *IS3000*, *IS5000*, and *IS8000* for DSI-derived SCs at maximum *bvals* of 3,000, 5,000, and 8,000, respectively, in Figure 8A) or corrected DTI-derived SCs (labeled *ISH3000*, *ISH5000*, and *ISH8000*). All these intrasubject errors were plotted against the pairwise mean absolute intersubject differences evaluated for DSI-derived SCs for each of the maximum *bval* (labeled *Inter3000*, *Inter5000*, and *Inter8000* in Figure 8A).

#### Fingerprinting potential.

The fingerprinting capability of the proposed model was first assessed on the SCs of the 42 test-retest subjects of the HCP-YA dataset, which were unrelated to the training data. The LBLR SCs of subjects scanned in Session 2 were corrected to produce HBHR SCs, and a distance matrix was then computed between Scan 1 HBHR SCs and Scan 2 SCs before or after correction (Figure 7C, *before harmonization* and *after harmonization*, respectively). The accuracy and I_diff_ were also calculated to evaluate the performance of the model. In addition, the intrasubject differences (in the form of L1 differences averaged across the connections) were calculated between the actual HBHR SCs of Session 1 and actual LBLR SCs of Session 2 (labeled *ISS1S2Diff* in Supporting Information Figure S3) or the corrected LBLR SCs of Session 2 (labeled *ISS1S2DiffHar*). These were plotted against the intersubject and intrasubject differences (labeled *InterS1S2* and *ISS1S2Same*, respectively) calculated between HBHR SCs of both sessions.

The fingerprinting capability of the proposed model was then assessed on the SCs of the 11 subjects of the Lausanne Psychosis Cohort dataset. A distance matrix was computed between DSI-derived SCs and DTI-derived SCs before and after correction (Figure 8BI, III, V, *before harmonization*, and Figure 8BII, IV, VI *after harmonization*). The accuracy and I_diff_ were calculated to evaluate the performance of the model for each of the *bvals*. In addition, the intrasubject differences (in the form of L1 differences averaged across the connections) were calculated between the DTI SCs corrected across a range of *bvals* and the DSI SCs at maximum *bval* of 3,000 (Supporting Information Figure S4). Furthermore, DSI-derived SCs and DTI-derived SCs before and after correction for maximal *bval* of 3,000 are displayed for each of the 11 subjects (Figures S5, S6, and S7 in the Supporting Information).

## RESULTS

### Effects of Acquisition Parameters

SCs before harmonization are displayed, for all combinations of APs and an indicative subject, in Figure 2. Regardless of the exact choice of parameters, all types of structural connections could be accurately resolved. In particular, short association fibers (U-fibers) across all cortical regions yielded the strongest signal. Long association fibers were also detected, such as fronto-temporal or fronto-parietal connections, which likely respectively highlight the uncinate fasciculus or the superior longitudinal and arcuate fasciculi. Homotopic frontal and occipital connections were observed (commissural fibers), as well as cross-hemispheric connections between the basal ganglia and thalamic substructures. Furthermore, projection fibers were also detected, particularly involving the thalamo-cortical, cortico-spinal, and cortico-pontine tracts, as well as connections between the basal ganglia and the cortex.

Upon statistical analysis at the population level, carried out independently for each connection (Figure 3 and Table 1A), *bval* was the factor that yielded the highest number of significant outcomes (percentage of connections significantly affected by the AP: 24%), followed by *res* (5%) and their interaction (0.88%). All types of fibers were concerned. For the effect of *bval* as well as its interaction with *res, p* values were noticeably low and widespread across subcomponents from the basal ganglia. At the level of graph theoretical metrics (Figure 4 and Supporting Information Figure S1), for nodal strength and closeness centrality (reflective of integration), significance was broadly distributed (percentage of regions reaching significance: 100/100%, 80/55%, and 61/66% for the effects of *bval*, *res*, and their interaction, respectively), and the strongest for *bval*. Regional involvement was quite uniform for closeness centrality, while for nodal strength, frontal, insular, and limbic regions as well as deep gray nuclei dominated. For clustering coefficient and local efficiency (reflective of segregation), the opposite was observed: the broad pattern of significance (69/62%, 93/95%, and 57/78% of regions reaching significance) was this time the strongest for *res* overall. The only exception was the basal ganglia, where significance was stronger for *bval*.

SCs after harmonization on the same indicative subject as in Figure 2 are displayed, for all combinations of APs, in Figure 5. Overall, harmonization was effective: Comparing the outputs across *res* with fixed *bval*, no qualitative differences were observed, and upon ANOVA on the test set, the impact of *res* on edges decreased from 0.40% to only 0.19% of significant connections (Table 1B). Comparing the outputs across *bval* with fixed *res*, while the overall pattern was similar, structural connectivity was overestimated for a subset of connections with originally small values, resulting in a denser looking output. The impact of *bval* on the test set even decreased from 8% to 0.46% (Table 1B). However, the number of significant edges affected by the interaction between factors increased from 0.01% to 0.24% after correction (Table 1B). When investigating harmonization at the level of graph theoretical metrics (Figures 6A–6D and Table 1B; see also Supporting Information Figure S2), for nodal strength and closeness centrality, the originally present difference across the factors in the test set was almost corrected, reflecting efficient harmonization; while for clustering coefficient and local efficiency, before harmonization, an effect of *res*, as well as an interaction between factors, could be observed, while after harmonization, they were almost removed while an effect of *bval* appeared. In a two-dimensional summary space in which the data before and after harmonization were projected using PCA (Figure 6E), before correction, the first principal direction encoded *bval*, and the second *res*, with clearly separable clouds of data between all parameter subcases. After correction, separation became hardly possible, and the first two principal directions both encoded connection patterns representative of differences across *bval*, respectively for a subset of subjects (first dimension) or the whole group (second dimension).

### Quality of Harmonization

To quantify the quality of harmonization, the distances between subject-wise connectivity patterns at (1.25, 3k) and those harmonized from (2.3, 1k) to (1.25, 3k) (that is, the errors following harmonization) were computed, on the test data, when training the model with various settings (Figure 7A). These distances were compared with (a) the cross-subject distances for connectivity patterns at (1.25, 3k) (labeled *InterS* in Figure 7A, an upper bound for fingerprinting to be achievable with harmonization) and (b) the distances between subject-wise connectivity patterns at (1.25, 3k) and (2.3, 1k) (a measure of the original distance induced by parameter differences; labeled *IS*). The KS test revealed no significant difference between the *InterS* and *IS* distributions (*p* value = 0.22). However, after harmonization, regardless of the training settings, the reconstruction errors (labeled *IS150*, *IS75*, *IS50*, *IS25*, *IS5*, *IS150M*), even though they visibly increased when using 5 TS (*IS5*) (20 data points) rather than 25 TS (*IS25*) (100 data points), were consistently smaller than *InterS*. Reconstruction errors were unchanged by the use of 150 independent data points (*IS150M*; that is, one parameter subcase for 150 different subjects) compared with the use of 25 TS (*IS25*; that is, 100 training data points in total). Also, for this model (trained using 150 independent data points), the intrasubject differences (*IS150M*) were still lower and significantly different (*p* value < 0.05 for the KS test) than the intersubject differences (labeled *InterS150M*).

To showcase the generalization potential of the model, reconstruction errors were computed when estimating SCs at (2, 2k) from (2.3, 1k), and compared with the same quantities as above (Figure 7B). Intersubject distances were higher than intrasubject ones, signifying that the impact of parameter differences was mild, but reconstruction errors were even smaller, demonstrating that the model can be efficiently generalized to parameter values unseen in training. This was accompanied by an I_diff_ increase from 2.10 to 2.52, denoting an increase in the distance between average intersubject and intrasubject distances in connectivity patterns.

To more directly assess fingerprinting potential, fingerprinting accuracy of the model trained on 150 TS was quantified on 42 independent TRT subjects when matching one scan at (1.25, 3k) with another at (2.3, 1k) (accuracy before harmonization), or one scan at (1.25, 3k) with the other harmonized from (2.3, 1k) (accuracy after harmonization; Figure 7C). Accuracy increased from 88% to 100% upon harmonization, while I_diff_ increased from 1.37 to 1.99, denoting an increase in the distance between average inter- and intrasubject distances in connectivity patterns. This can be qualitatively validated from Supporting Information Figure S3, where the intrasubject differences across APs across sessions before harmonization (labeled *ISS1S2Diff*) seem on par with the intersubject differences at the APs (1.25, 3k) across sessions (labeled *InterS1S2*), whereas after harmonization, these intrasubject differences across APs across sessions (labeled *ISS1S2DiffHar*) seem to be within the upper bound set by the minimum of the *InterS1S2* and not lower than the intrasubject differences across sessions at the same APs (labeled *ISS1S2Same*).

Finally, to evaluate the generalization capacity of the model, reconstruction errors were also computed when estimating DSI-derived SCs from the DTI-derived SCs on the 11 subjects of the Lausanne Psychosis Cohort. For DSI at maximum *bval* = 3,000 and 5,000, these reconstruction errors (labeled as *IS3000, IS5000* in Figure 8A) decreased after harmonization (labeled as *ISH3000*, *ISH5000*) and became smaller than the respective intersubject distances (labeled as *Inter3000, Inter5000*). However, the opposite was observed for DSI at maximum *bval* = 8,000. The reconstruction error (labeled as *IS8000*) increased after harmonization (labeled as *ISH8000*) and became larger than the intersubject distances (labeled as *Inter8000*). Similar observations were made in the distance matrices computed at these maximum *bval* settings. Intrasubject distances (diagonal elements in the matrices from Figure 8BII, IV) were the lowest for all but one subject for DSI data at maximum *bval* = 3,000 and 5,000 after correction. These results were quantitatively validated by an increase in the accuracy of fingerprinting subjects from 72% to 90% and from 81% to 90% and an increase in I_diff_ from 1.38 to 1.57 and from 1.39 to 1.53 upon harmonization for maximum *bval* = 3,000 and 5,000, respectively. However, at maximum *bval* = 8,000, even though the accuracy of fingerprinting increased from 72% to 81%, the I_diff_ decreased from 1.34 to 1.16. This could also be seen qualitatively from Figure 8BV, VI, where the differences between the intersubject and intrasubject distances were less pronounced after harmonization.

## DISCUSSION

Large-scale multisite studies are important to discover neuroimaging biomarkers for many diseases, such as Alzheimer’s, Parkinson’s, or multiple sclerosis, just to cite a few, which otherwise would remain underpowered considering small effect sizes (Thompson et al., 2014). However, aggregating data from several centers adds site-specific biases that need to be controlled for. Even in the case of optimal alignment of APs across sites, important differences can remain (Cai et al., 2021; Radua et al., 2020). Ways to harmonize heterogeneous datasets via postprocessing have been proposed (Wachinger, Rieckmann, & Pölsterl, 2021), one of the most popular approaches of harmonization being ComBat. While ComBat has demonstrated its efficacy even at the level of structural connectivity matrices (Onicas et al., 2022), the need to explicitly model biological covariates can be problematic under certain conditions (Bayer et al., 2022). On well-balanced categorical datasets, such as patient-control studies, ComBat and the like perform well (Bayer et al., 2022). However, ComBat is not suited to harmonize data in a more general context, without a specific case-control setting. For example, in tasks like fingerprinting subjects based on their connectomes, it is impossible to qualify all the biological covariates relevant to retain interindividual specificity.

Other approaches that correct the bias at the raw DWI level using rotation invariant harmonic features (RISH) (Karayumak et al., 2019) may be relevant in this context, but these approaches have been developed to harmonize images at the voxel level, not on connectivity. Besides, the correction of the bias at the voxel level using approaches like RISH may be helpful to harmonize the DWI scalar maps, but when it comes to SCs, any small difference in position or ODF due to registration problems might be propagated and exaggerated on the tractography process and finally the connectome (Kurokawa et al., 2021). Recent developments using deep learning to retain scanner invariant information using domain adaptation (Dinsdale, Jenkinson, & Namburete, 2020, 2021) reported mitigated results, suffering from the “worst-scanner syndrome” (Moyer & Golland, 2021).

As opposed to ComBat, which models site and subject category, we took the path of learning the statistical effects on the images of specific APs. To do so in a controlled environment where the optimum is known, we created a resampled dataset from the HCP-YA dataset, with two known sources of variation, namely *b*-value and spatial resolution, while keeping everything else constant.

Our analysis of variance revealed that *b*-value and spatial resolution were two independent parameters influencing brain connectivity, with only little interaction. Similar effects were reported by Papinutto, Maule, and Jovicich (2013) at the level of fractional anisotropy and mean diffusivity.

From there, we took a linear regression approach to correct for these acquisition biases. Since we had four AP combinations in our resampled dataset, each combination can be considered one site. However, instead of using these sites as categorical covariates, the factors of variation (i.e., the APs and their interaction) were used as regression covariates for predicting the connection strength between two brain regions. In short, each connection was modeled as a linear combination of the APs and their interaction. Also, it was designed to work in a machine learning framework, and hence no explicit modeling of subject-specific covariates was performed.

### Effects of Acquisition Parameters

As we could see from Figures 2 to 4, there is a clear impact of APs on SCs and the derived graph metrics.

It seems that the variation of *b*-value, spatial resolution, and their interaction generates different types of biases on the SCs. Crossing fibers, which are highly prevalent in the deep white matter (around 30% to 90% according to K. G. Schilling et al., 2022), and highly curved subcortical connections, may be particularly sensitive to APs.

As seen from Figure 3 and Table 1, the *b*-value has the highest impact on the number of edges, and this impact is spread out on most of the connections. This may be due to the prevalence of crossing fibers throughout the white matter and the known dependence between *b*-value and ODF sharpness (K. G. Schilling et al., 2017). High *b*-value and angular resolution are essential to map crossing fibers (Tuch et al., 2002). The spatial resolution also plays an important role but seems to affect predominantly U-fiber connectivity. U-fibers follow the cortex and hence exhibit high curvature. Those sharp turns may be resolved solely with sufficient spatial resolution. The least impacting factor on connectivity is the interaction between *b*-value and spatial resolution, which is nonetheless significant for connections related to the basal ganglia and the thalamus. These structures are located deep inside the brain. Their connections have to cross major white matter tracts such as the internal capsule and the centrum semi-ovale, and connections have a wide distribution of length (short intrinsic connectivity, long range to the cortex). It may be that resolving those connections is particularly demanding in terms of acquisition quality where not only *b*-value but also spatial resolution plays a key role (K. Schilling et al., 2017). It may also be that the interaction arises from the necessity to have at least a decent combination of APs, either high *b*-value or high spatial resolution or a combination of both.

As seen from Figure 4 and Table 1, APs impact individual graph metrics differently. On a broad scale, we see that the *b*-value has the highest impact on measures of integration (i.e., nodal strength and closeness centrality), whereas spatial resolution has the highest impact on segregation measures (i.e., clustering coefficient and local efficiency).

Integrative measures are reflective of the overall efficiency of the brain and may be impacted by the loss of long association connections, which are important shortcuts to ensure low average shortest path length. As discussed in the above paragraph, the mapping of fiber crossings is particularly sensitive to *b*-value, thus leading to the loss of connections at low *b*-value, particularly in the deep white matter, which results in decreased nodal strength (Figure 6A) and decreased closeness centrality (Figure 6B). The effect of the *b*-value on nodal strength seems to be more regional, whereas it seems more widespread for closeness centrality. A possible reason might be that nodal strength depends on immediate connections whereas closeness centrality depends on the overall connection pattern in the network (Figure 3, Figures 4A, 4B).

Segregation measures capture local connectivity. Local connectivity is materialized by U-fibers. Their sharp bending below the cortex might only be resolved accurately if spatial resolution is sufficient. At insufficient spatial resolution, these fibers may get captured within these regions, thus forming a strong modular structure, which explains the higher clustering coefficient (Figure 6C) and higher local efficiency (Figure 6D) with lower spatial resolution. However, the effect of *b*-value is higher for these local measures in the basal ganglia. A possible reason might be the presence of multiple short- and long-fiber crossings in this region, which might have a higher dependency on *b*-value rather than spatial resolution.

An interesting observation was the difference in how strongly APs impacted structural connections compared with graph metrics (particularly nodal strength and closeness centrality), as much larger percentages of regions were involved in the latter case compared with edge percentages in the former (see Table 1). While the use of Bonferroni correction may partly explain this difference, as it neglects dependencies across individual tests in both cases but corrects more stringently for edges, the inner nature of graph metrics, which essentially combine information across several connections, is also likely to play a role. This is why integrative graph metrics are more impacted, since they draw on the full set of connections, while metrics reflective of segregation consider only a few.

Figure 5 showcases the SCs of the same indicative subject at the different APs after harmonization to HBHR. Overall, the harmonization was effective, as the effect of the APs is qualitatively reduced. Table 1B summarizes the effect of harmonization using a two-way ANOVA on the test set at the level of edge and node features. It can be seen that whereas the effect of *b*-value and spatial resolution at the level of edges seems to have been greatly attenuated, the effect of their interaction seems to have increased slightly. This might be due to the overestimation of number of fibers at HBHR by our model, which does not consider the decrease in signal-to-noise ratio at higher *b*-values and spatial resolution (Crater et al., 2022; K. Schilling et al., 2017). This might also explain the overestimated connections for certain brain regions when lower *b*-values (1,000 s/mm^2^) are corrected to higher *b*-values (3,000 s/mm^2^) in Figure 5.

From our results, we can conclude that overall, graph metrics are well corrected by our model (Figure 6 and Table 1B). The model, however, performs differently on integration and segregation measures depending on whether *b*-value, spatial resolution, or both are corrected.

Integration measures are well corrected in any harmonization scenario. Whereas segregation measures under different *b*-values are not well corrected with our model, correcting for spatial resolution works well. The overestimated fibers after harmonization in Figures 5C and 5D may not be reflected in the integration measures but may be captured by the local segregation measures.

The positive impact of harmonization can also be seen in the embedding space spanned by the top two principal components obtained using PCA (Figure 6E). The structural connectivity matrices of subjects at different APs separated from each other when projected onto this space, where the first dimension seems to naturally encode for *b*-value and the second dimension for spatial resolution. After harmonization, separate AP groups could no longer be distinguished from each other.

### Quality of Harmonization

As seen from Figure 7A, the intrasubject differences between the HBHR data and corrected LBLR data are at the level of intersubject variability (*p* value > 0.05 for the KS test), alleviating any possibility to distinguish AP effect from interindividual variability. After harmonization, the situation clearly improves since the mean L1 difference of intrasubject variability is divided by 2, while the interindividual variability remains at a higher level. This observation is true regardless of whether TS are used. This is important since, in practice, having TS is always related to additional organizational complexity and increased cost (Roffet et al., 2022). This result might be explained since the model did not use any subject-specific covariate, and hence the harmonization performance is more dependent on the total number of training points available (dependent or independent) rather than the number of TS. Taken together, these results shed light on the fact that even with the simplest linear approach, the acquisition bias can be minimized and fingerprinting is a viable goal.

We designed a model with continuous variables rather than categorical ones. This approach gives the opportunity to generate SCs for APs that were not included in the training set. In other words, we have a generative model. Since the difference between connectomes produced at IBIR and LBLR is not as high as comparing the extreme SCs produced at HBHR and LBLR, the decrease in intrasubject differences after harmonization is not that pronounced (Figures 7A and 7B). However, the efficacy of our model can be substantiated by the increase in I_diff_ from 2.10 to 2.52 after harmonization. This larger difference between intersubject and intrasubject distances may benefit classification models like support vector machines (Cortes & Vapnik, 1995), which tend to perform better when the margin between the different classes (here, SCs of different subjects) increases.

Also, using APs as regression covariates enabled, in some settings, efficient generalization of the model (trained on the HCP-YA data with *b*-values of 1,000 and 3,000) to a completely independent dataset containing different diffusion imaging acquisitions. Indeed, harmonization of DTI-derived SCs at *b*-value of 1,000 to SCs obtained with DSI acquisitions at maximum *b*-values of 3,000 and 5,000 could be successfully achieved. However, harmonization to acquisitions at a maximum *b*-value of 8,000 was less satisfying (Figure 8A). This means that our model was good enough to extrapolate within a specific range, but then failed for higher *b*-values because of their nonlinear effect, resulting in an overestimation of structural connectivity at the highest examined *b*-value. Furthermore, another result speaking in favor of our model’s efficacy is the mean intrasubject distance when DSI-derived SCs at maximum *b*-value of 3,000 were compared with the DTI-derived SCs harmonized to various *b*-values between 1,250 and 5,000, as the minimum occurred near 3,000, as should be expected from an unbiased model (Supporting Information Figure S4).

The goal of any such harmonization is to perform analyses that may include discovering biomarkers for a particular disease (Catani, 2009), predicting brain age, or maybe even tasks like identifying individuals based on their connectomes (Badhwar et al., 2020). As seen from Figure 7C, after correcting the LBLR SCs of the second session, they matched with the HBHR SCs of the first session at the highest level, as reflected by an increased harmonization accuracy of fingerprinting from 88% to 100%. For the Lausanne Psychosis Cohort and DSI maximum *b*-value settings of 3,000 and 5,000, all but one subject could be identified when each DSI-derived SC was compared against the harmonized DTI-derived SCs (Figure 8B). The one subject with less successful fingerprinting showed a quite dense DTI-derived SC compared with others for the same setting, which was thus closer to the DSI-derived SC at maximum *b*-value of 3,000 before harmonization. For this reason, our model overestimated the *b*-value effect for this particular subject (Supporting Information Figure S5). For DSI at maximum *b*-value of 8,000, even though the accuracy of fingerprinting improved, the difference between the intersubject and intrasubject distances became less pronounced after harmonization (Figure 8B), denoting an unstable model. In sum, our harmonization procedure is capable of conserving subject-specific variability while removing the acquisition bias, except in the specific cases of characteristically distinct subjects or of estimations performed at parameter values outside of the training range, if nonlinearity is present.

### Limitations and Future Work

Despite its good performance, our model also has some limitations. Here, even though we do not need to model for the biological covariates, the model needs to be informed about acquisition-related parameters. In our case, these parameters are well known, namely *b*-value and spatial resolution. However, in a “real-life” multisite setting, this may not be the case. When several scanner models are used, maybe even from different vendors and with different pieces of equipment, it may become impossible to have an exhaustive list of sources of variation. In this setting, it may be wise to use surrogate variable analysis (Fortin et al., 2017) or PCA (Wachinger et al., 2021) across all the image features on the whole dataset to detect the unknown sources of scanner variation and then build a model on this basis. Another approach could be using image quality metrics and metadata (Carré et al., 2022) to assign batch labels and then use predictive probabilities provided by Gaussian processes as covariates for these batch labels to regress out the site effects instead of using simple one-hot encoded covariates (Monte-Rubio et al., 2022). Also, another limitation of our model is that it used data from healthy subjects within the age range of 22 and 36. Hence, it should next be tested with more heterogenous data, including the potential presence of brain disorders and a wider age range. One possible way of accounting for this will be to include a regression covariate (linear or nonlinear as in Pomponio et al., 2020) for age and its interaction with each of the APs, as well as categorical covariates for each of the brain disorders and their interactions with each of the APs. A more realistic setting of case-control multisite studies could be modeled similarly except that the covariates would include all the APs used in the sites for producing the scans. These APs might be obtained from documentation, or by using the aforementioned methods on the data of healthy subjects to find the unknown sources of scanner variations. The validation of our model with only 42 TRT data is quite small to be of clinical relevance. Hence, validation in a larger sample size is a prospect for future studies.

Finally, our model is overcorrecting the impact of *b*-value, as it is undermining the impact of the decrease in signal-to-noise ratio for HBHR SCs, which might be a disadvantage for extrapolating the model to higher *b*-values. This may be solved by either modeling this impact directly or introducing some form of nonlinearity or regularization in our harmonization model. The nonlinear modeling of certain covariates as with the ComBat generalized additive model (Pomponio et al., 2020; Sun et al., 2022) or the modeling of the effect of bias on the covariance between different features (edges) like with CovBat (Chen et al., 2022) could be considered. L1 or L2 regularization can be added to the linear regression, or some form of geometry-constrained harmonization (Simeon, Piella, Camara, & Pareto, 2022) might also be helpful. In order to add all of these together, a graph variational auto-encoder (Kipf & Welling, 2016) could be applied to capture the dependency between the SCs and project them to an embedding space where the factors of variation could be disentangled.

## CONCLUSION

In this study, we explored the effect of acquisition bias (i.e., *b*-value and spatial resolution) on the SCs, making sure that all other factors were constant. The effect of *b*-value was much more pronounced on the edges, embedding space, and the graph metrics reflective of integration, whereas the impact of spatial resolution predominantly affected segregation measures. Our proposed linear regression machine learning model was successful in removing this acquisition bias even without the use of TS. Also, subjects could be correctly fingerprinted at a high level even when they were acquired with different APs thanks to our correction method. Noteworthily, owing to its formulation, our model can not only generate SCs at different *b*-values and spatial resolutions for unseen subjects, but also showed its ability to generalize to a fully independent dataset reflective of typical clinical acquisition practices. Taken together, these results shed light on the fact that even with the simplest linear approach, acquisition bias can be minimized, making fingerprinting possible across heavily biased connectomes. This yields great potential for multicentric, large-scale studies.

## ACKNOWLEDGMENTS

This work has been financially supported by the Swiss National Science Foundation grant number 197787.

## SUPPORTING INFORMATION

Supporting information for this article is available at https://doi.org/10.1162/netn_a_00368.

## AUTHOR CONTRIBUTIONS

Jagruti Patel: Conceptualization; Data curation; Formal analysis; Investigation; Methodology; Software; Visualization; Writing – original draft; Writing – review & editing. Mikkel Schöttner: Conceptualization; Writing – review & editing. Anjali Tarun: Conceptualization; Data curation; Supervision. Sebastien Tourbier: Data curation; Software; Supervision; Writing – review & editing. Yasser Alemán-Gómez: Data curation; Writing – review & editing. Patric Hagmann: Conceptualization; Funding acquisition; Investigation; Methodology; Project administration; Resources; Supervision; Validation; Writing – review & editing. Thomas A. W. Bolton: Conceptualization; Formal analysis; Investigation; Methodology; Supervision; Validation; Visualization; Writing – review & editing.

## FUNDING INFORMATION

Patric Hagmann, Schweizerischer Nationalfonds zur Förderung der Wissenschaftlichen Forschung (https://dx.doi.org/10.13039/501100001711), Award ID: 197787.

## Supplementary Material



## TECHNICAL TERMS

Fractional anisotropy: The degree of anisotropy of water diffusion within a voxel or a region of interest.
Mean diffusivity: The average amount of diffusion-related water mobility within a voxel or a region of interest.
Tractography: A technique used to map white matter axonal trajectories in vivo and noninvasively using diffusion MRI.
*b*-value: An MRI-related parameter defining the level of diffusion sensitivity in the image.
Fixed effect: A variable whose effect is constant across individuals.
Random effect: A variable whose effect varies across individuals.
Constrained spherical deconvolution: A method to reconstruct diffusion-weighted images in order to perform downstream tractography.
Orientation distribution function: A function defined on a sphere informing the diffusion speed in different directions.
Two-way ANOVA: A statistical test used to assess the effect of two independent variables and their interaction on a dependent variable.
Principal component analysis: It transforms high-dimensional data to low-dimensional data while preserving maximum variation.
Standardized: The values are scaled to have a mean of zero and a unit standard deviation.
Categorical covariates: Covariates that are categorical independent variables in a regression model.
Regression covariates: Covariates that are continuous independent variables in a regression model.
