# Supplementary material for: Modeling the impact of MRI acquisition bias on structural connectomes: Harmonizing structural connectomes
Source: Netw Neurosci. 2024 Oct 1;8(3):623–52. doi: 10.1162/netn_a_00368 (PMC11340995; doi:10.1162/netn_a_00368)
Supplement: Supplementary file 1 [file netn-8-3-623-s001.pdf]

Journal: **NETWORK NEUROSCIENCE**

SUPPLEMENTARY

Modeling the impact of MRI acquisition bias on structural connectomes: Harmonizing structural connectomes

Jagruti Patel[1], Mikkel Schöttner[1], Anjali Tarun[1], Sebastien Tourbier[1], Yasser Alemán-Gómez[1], Patric Hagmann[1], and Thomas A. W. Bolton[1]

[1] Department of Radiology, Lausanne University Hospital and University of Lausanne (CHUV-UNIL), Lausanne, Switzerland

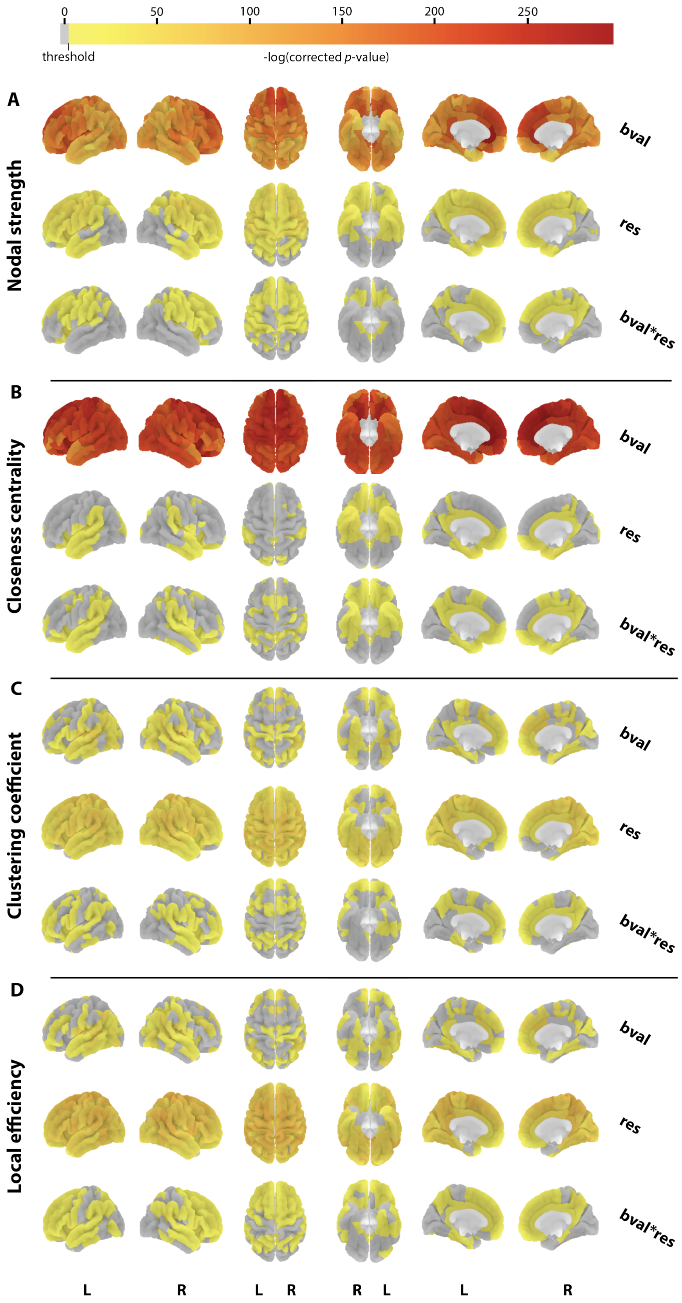

Figure S1. Impact of acquisition parameters on nodal metrics extracted from whole-brain structural connectivity.

Surface plots to show the results of 2-way ANOVA in terms of the impact of b-value (bval), spatial resolution (res), and interaction between these Aquisition Parameters (AP) (bval*res), on (A) nodal strength, (B) closeness centrality, (C) clustering coefficient and (D) local efficiency. The ANOVA analysis was done independently on each of the nodes for each of these metrics across the entire dataset (training and test) of 190 subjects for the 4 AP combinations. Each value in the surface plot is the negative of the log of the Bonferroni-corrected p-values (p-value $\times$ 274, where 274 is the number of nodes). Any value lower than or equal to the threshold (-log(0.05)) is insignificant (gray in colour). Any value larger than this threshold is significant and the redder the colour, the larger the significance of the bias on a node for the corresponding nodal metric. [Note: 'L' stands for left and 'R' stands for right.]

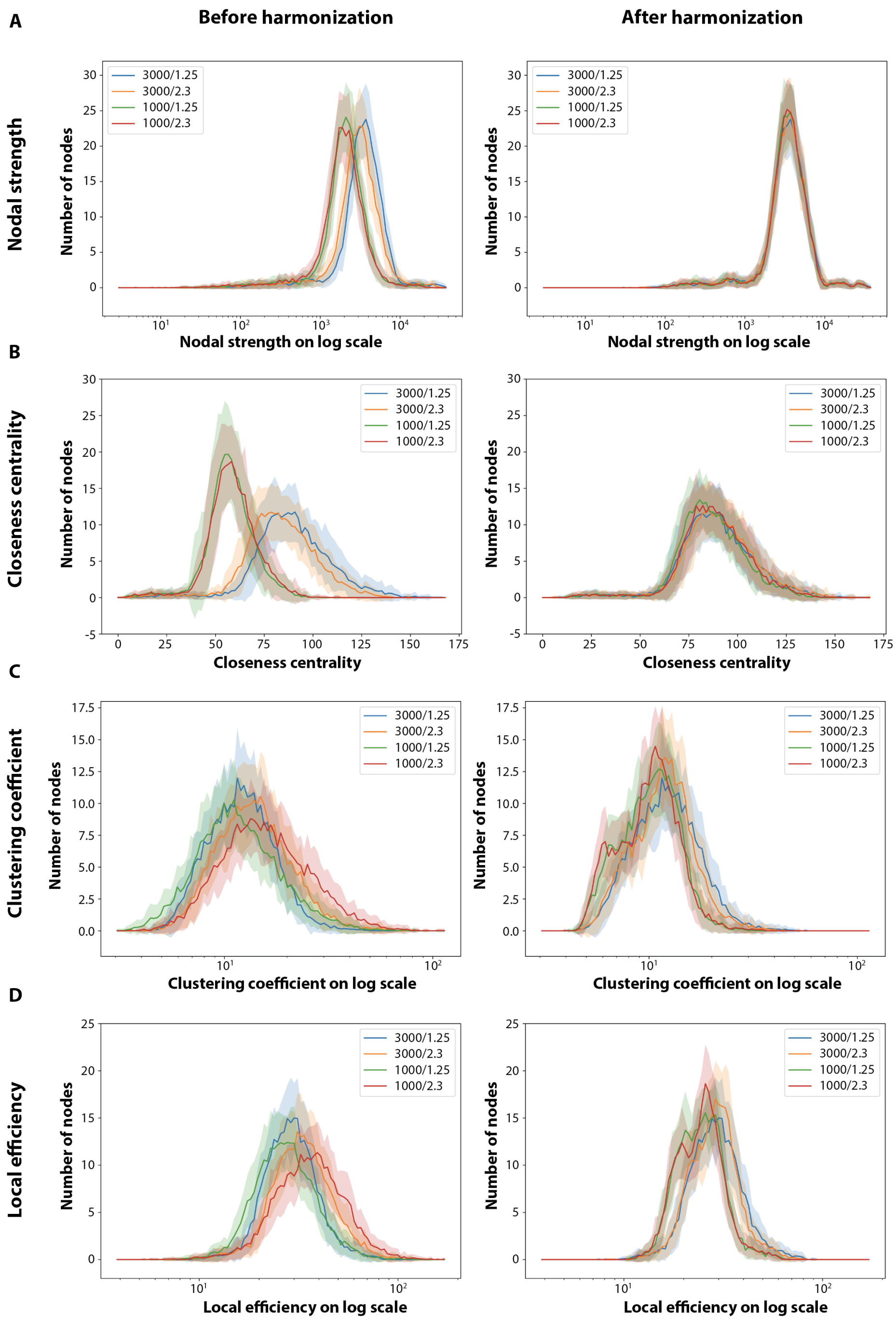

Figure S2. Effect of harmonization on the graph metrics.

Histograms for (A) nodal strength, (B) closeness centrality, (C) clustering coefficient and (D) local efficiency computed for the 40 test subjects across the 4 Acquisition Parameter (AP) combinations (3000/1.25 for b-value (bval)=3000, spatial resolution (res)=1.25; 3000/2.3 for bval=3000, res=2.3; 1000/1.25 for bval=1000, res=1.25; 1000/2.3 for bval=1000, res=2.3). All the plots on the left are before harmonization and the ones on the right are after harmonization. [Note: The thick line represents the mean and the error surface represents standard deviation.]

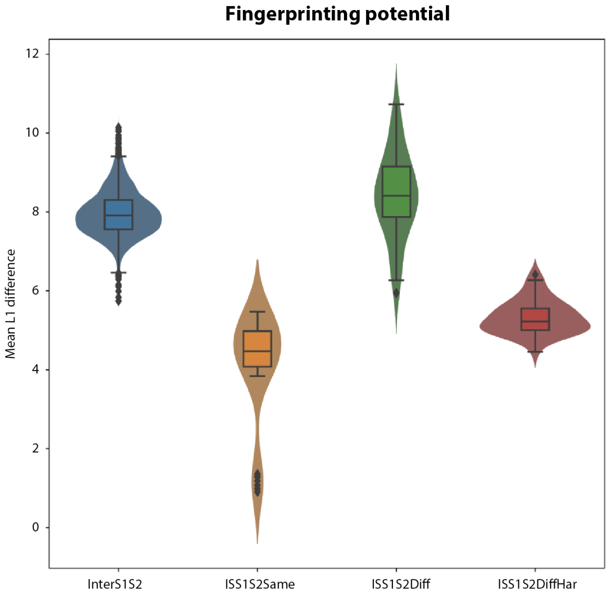

Figure S3. Fingerprinting potential.

The distribution of mean L1 differences across test subjects across scans [at Acquisition Parameter (AP) (1.25, 3k), InterS1S2] is compared to that within subjects across scans [at AP (1.25, 3k), ISS1S2Same], across APs across scans before harmonization [(1.25, 3k) versus (2.3,1k), ISS1S2Diff], and after harmonization [(1.25, 3k) versus (2.3, 1k) corrected (ISS1S2DiffHar) using 150 traveling subjects in model fitting (i.e., each contributing four data points)].

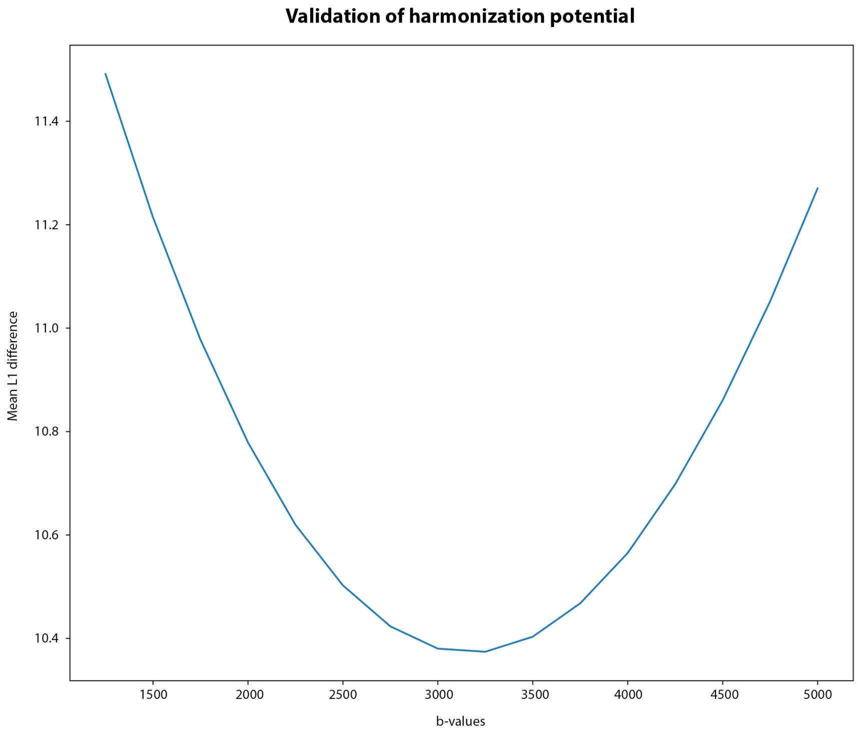

Figure S4. Harmonization quality across different *b*-values.

Mean intra-subject L1 difference when the diffusion tensor imaging-derived structural connectomes (SCs) of the 11 subjects of the Lausanne Psychosis Cohort, corrected to a range of *b*-values (*bval*, X-axis) from 1250 to 5000 and to a spatial resolution similar to the diffusion spectrum imaging (DSI) one, are compared against the DSI-derived SCs at a maximum *bval* of 3000. Correction was performed using the linear regression model trained on 150 traveling subjects (i.e., each contributing four data points) of the Human Connectome Project Young Adult dataset.

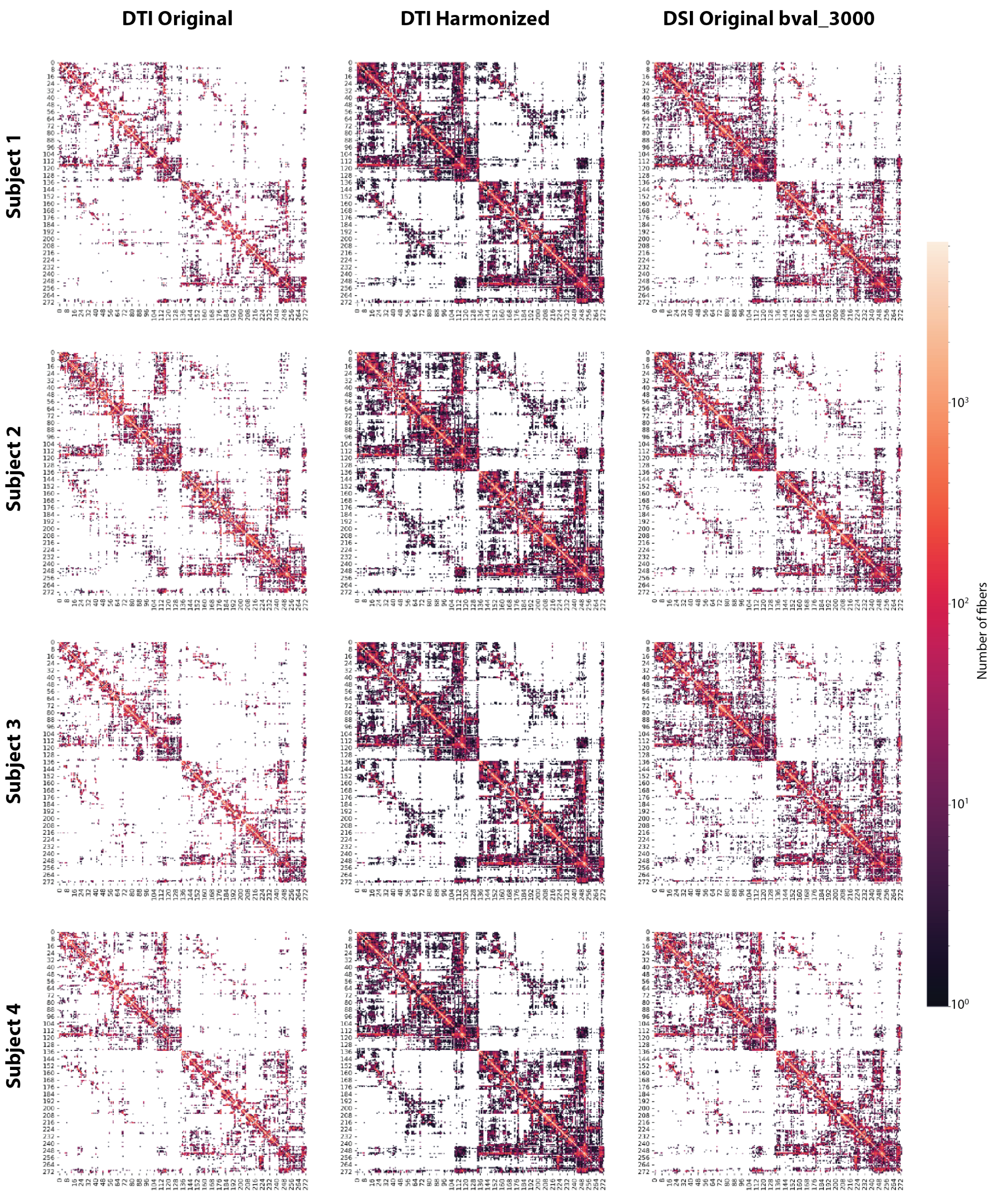

Figure S5. Structural connectomes before and after correction across the different diffusion acquisitions (1).

The first column shows the diffusion tensor imaging (DTI)-derived structural connectomes (SCs) of subjects 1 to 4 of the Lausanne Psychosis Cohort. The second column shows their DTI-derived SCs corrected to the diffusion spectrum imaging (DSI)-derived SCs at a maximum *b*-value (*bval*) of 3000. The third column shows their DSI-derived SCs at maximum *bval* of 3000. The correction was done using the linear regression model trained on 150 traveling subjects (i.e., each contributing four data points) of the Human Connectome Project Young Adult dataset.

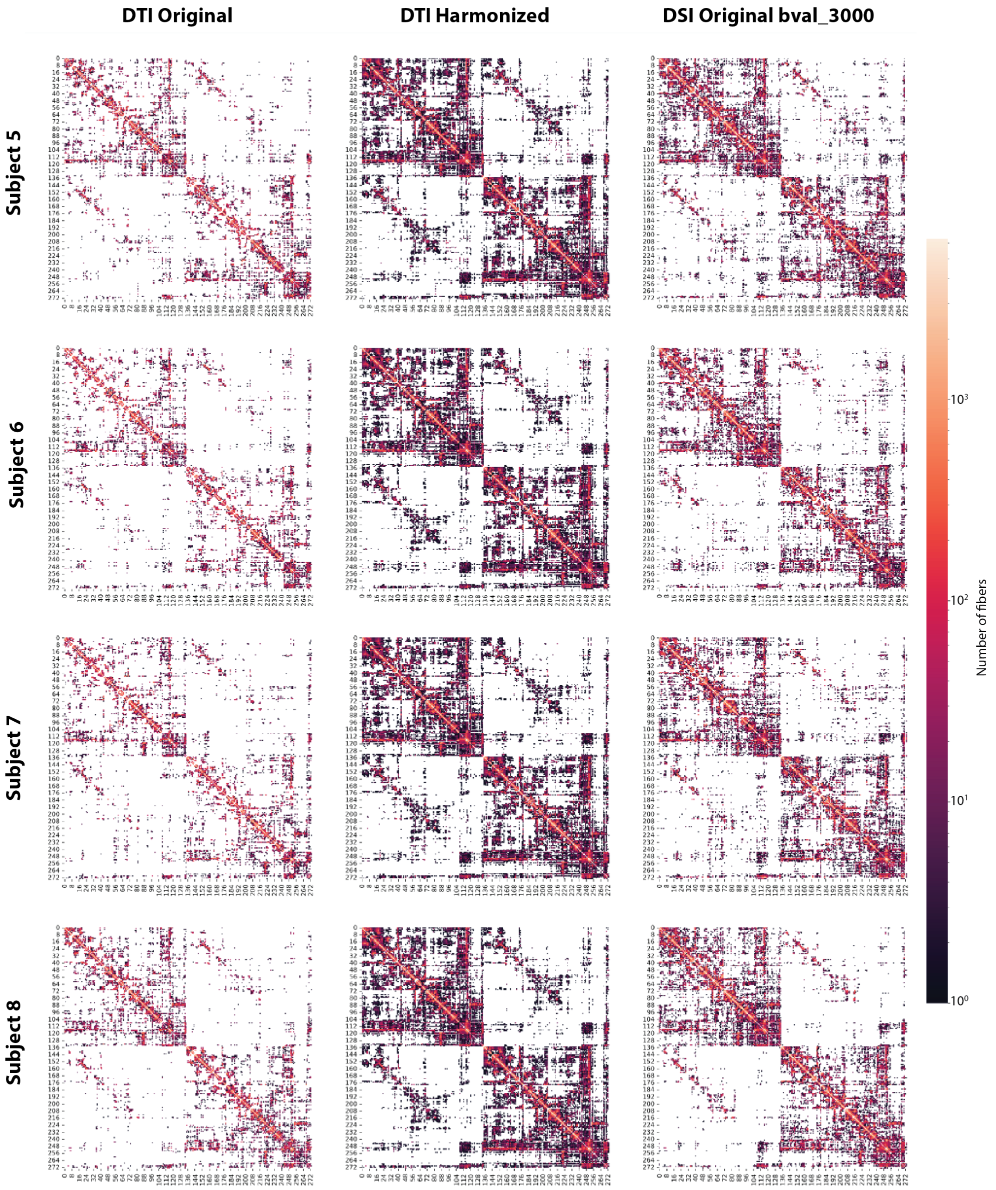

Figure S6. Structural connectomes before and after correction across the different diffusion acquisitions (2).

The first column shows the diffusion tensor imaging (DTI)-derived structural connectomes (SCs) of subjects 5 to 8 of the Lausanne Psychosis Cohort. The second column shows their DTI-derived SCs corrected to the diffusion spectrum imaging (DSI)-derived SCs at a maximum b-value (*bval*) of 3000. The third column shows their DSI-derived SCs at maximum *bval* of 3000. The correction was done using the linear regression model trained on 150 traveling subjects (i.e., each contributing four data points) of the Human Connectome Project Young Adult dataset.

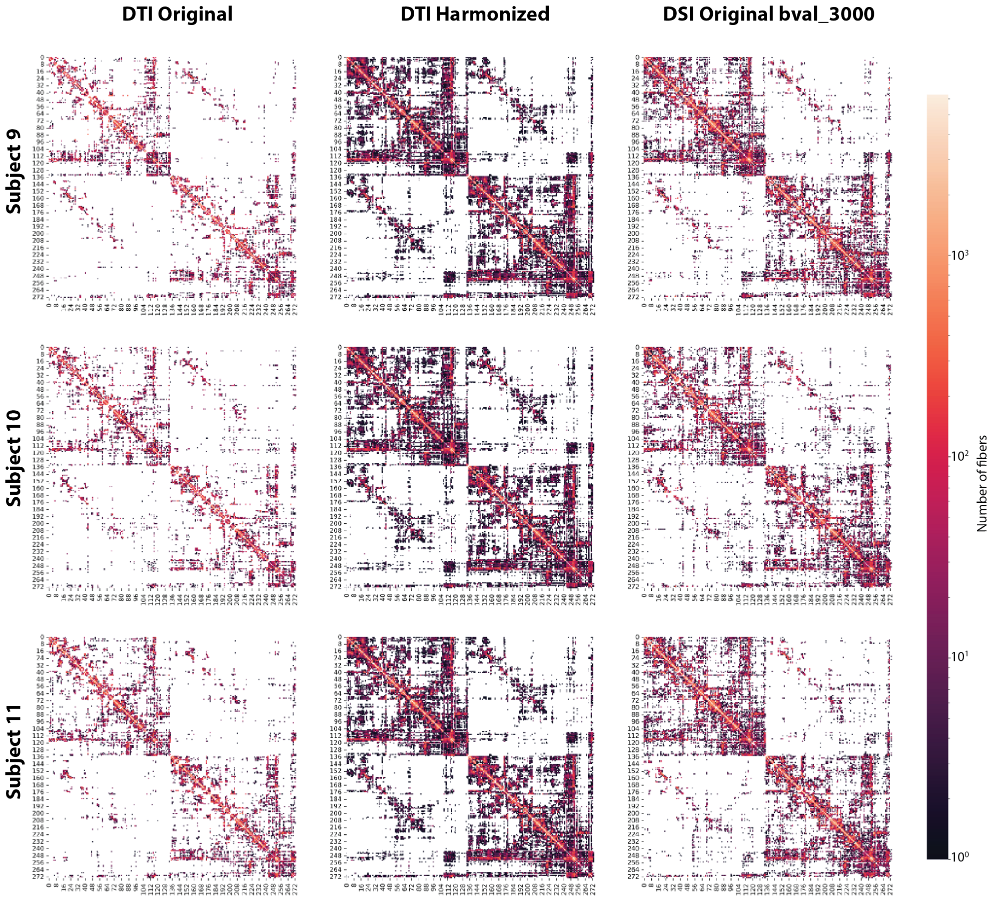

Figure S7. Structural connectomes before and after correction across the different diffusion acquisitions (3).

The first column shows the diffusion tensor imaging (DTI)-derived structural connectomes (SCs) of subjects 9 to 11 of the Lausanne Psychosis Cohort. The second column shows their DTI-derived SCs corrected to the diffusion spectrum imaging (DSI)-derived SCs at a maximum *b*-value (*bval*) of 3000. The third column shows their DSI-derived SCs at maximum *bval* of 3000. The correction was done using the linear regression model trained on 150 traveling subjects (i.e., each contributing four data points) of the Human Connectome Project Young Adult dataset.
